# Longitudinal analysis of lifestyle risk factors, nutrition status and drivers of food choice among urban migrants in Ulaanbaatar, Mongolia, and Almaty, Kazakhstan: a formative study

**DOI:** 10.1017/S136898002400243X

**Published:** 2024-12-03

**Authors:** Sabri Bromage, Shamil Tazhibayev, Xin Zhou, Chang Liu, Enkhtsetseg Tserenkhuu, Oksana Dolmatova, Munkhbat Khishignemekh, Leyla Musurepova, Wusigale, Soninkhishig Tsolmon, Enkhjargal Tsendjav, Davaasambuu Enkhmaa, Rajesh Kumar Rai, Bayarmaa Enkhbat, Bilige Menghe, Davaasambuu Ganmaa

**Affiliations:** 1 Community Nutrition Unit, Institute of Nutrition, Mahidol University, 999 Phutthamonthon 4 Road, Salaya, Phutthamonthon, Nakhon Pathom 73170, Thailand; 2 Department of Nutrition, Harvard T.H. Chan School of Public Health, 655 Huntington Avenue, Building 2, Boston, MA 02115, United States of America; 3 Department of Micronutrients, Kazakh Academy of Nutrition, 66 Klochkov Street, Almaty 050008, Kazakhstan; 4 Department of Biostatistics, Yale School of Public Health, 60 College Street, New Haven, CT 06520-0834, United States of America; 5 Mongolian Health Initiative, Royal Plaza, Bayanzurkh District, Ulaanbaatar 13312, Mongolia; 6 Key Laboratory of Dairy Biotechnology and Engineering, Ministry of Education, Inner Mongolia Agricultural University, Hohhot 010018, China; 7 Tana Lab, Graduate School of Business, Mongolian University of Science and Technology, 8th Khoroo, Baga Toiruu 34, Sukhbaatar District, Ulaanbaatar 14191, Mongolia; 8 National Center for Maternal and Child Health, Khuvisgalchdin Street, Bayangol District, Ulaanbaatar 16060, Mongolia; 9 Human Nutrition Unit, Institute of Nutrition, Mahidol University, 999 Phutthamonthon 4 Road, Salaya, Phutthamonthon, Nakhon Pathom 73170, Thailand; 10 Department of Global Health and Population, Harvard T.H. Chan School of Public Health, 665 Huntington Avenue, Building 1, Boston, MA 02115, United States of America; 11 Department of Pathology & Forensic Medicine, School of Biomedicine, Mongolian National University of Medical Sciences, S. Zorig Street, Ulaanbaatar 14210, Mongolia; 12 Department of Pathology, Mongolia-Japan Hospital, Mongolian National University of Medical Sciences, Baruun Janjin 25 573, Ulaanbaatar 13270, Mongolia; 13 Channing Division of Network Medicine, Department of Medicine, Brigham and Women’s Hospital and Harvard Medical School, 181 Longwood Avenue, Boston, MA 02115, United States of America

**Keywords:** Urban migration, Acculturation, Food environment, Drivers of food choice, Nutrition transition, Nutritional epidemiology, Central Asia

## Abstract

**Objective:**

To quantify and compare concurrent within-person trends in lifestyle risks, nutrition status and drivers of food choice among urban migrants in Central Asia.

**Design:**

We collected panel data on household structure, drivers of food choice, nutrition knowledge and diverse measures of nutrition status and lifestyle risk from urban migrants at 0, 3, 6 and 9 months using harmonised methodology in two cities. Trends were analysed using mixed-effects models and qualitatively compared within and between cities.

**Setting:**

Ulaanbaatar, Mongolia, and Almaty, Kazakhstan.

**Participants:**

200 adults (22–55 years) who migrated to these cities within the past 2 years.

**Results:**

Adjusting for age and sex, each month since migration was positively associated with fasting TAG in Almaty (0·55 mg/dl; 95 % CI: 0·13, 0·94) and BMI (0·04 kg/m^2^; 95 % CI: 0·01, 0·07), body fat (0·14 %; 95 % CI: 0·01, 0·26) and fasting glucose (0·04 mmol/l; 95 % CI: 0·02, 0·05) and lipids in Ulaanbaatar (*P* < 0·05). In Almaty, nutrition knowledge (measured using an objective 20-point scale) declined despite improvements in diet quality (measured by Prime Diet Quality Score). The influence of food availability, price and taste on food choice increased in Almaty (*P* < 0·05). Upon multivariable adjustment, nutrition knowledge was positively associated with diet quality in Almaty and adherence to ‘acculturated’ diet patterns in both cities (*P* < 0·05). Different trends in smoking, sleep quality and generalised anxiety were observed between cities.

**Conclusions:**

Findings indicate heterogeneous shifts in nutrition, lifestyles and drivers of food choice among urban migrants in Central Asia and provide an evidence base for focused research and advocacy to promote healthy diets and enable nutrition-sensitive food environments.

Migration is a major driver of change in food cultures and systems globally^(1,2)^. Migrants bring traditional tastes and recipes to their new homes where they assimilate new habits, and this intersection affects supply and demand for foods among migrant and host populations^(3)^. Migrants are exposed to new languages, food environments and socio-economic circumstances that influence their awareness of the culinary uses and nutritiousness of foods, ability to access or afford foods, responses to food marketing and diets^(4–7)^. Shifts in migrants’ diets affect their nutrition status, which is also affected by migration-induced shifts in time use including adoption of more or less sedentary lifestyles and changes in sleeping patterns^(8,9)^.

Studies in diverse populations have documented changes in diets, lifestyles and nutrition accompanying migration, particularly international migration, and have posited or adapted frameworks of food choice in the context of acculturation to explain these changes^(10,11)^. Little research has examined how drivers of food choice (DoFC) change following migration, how these changes relate to trends in nutrition status and risk factors for nutritional disease and how these trends and relationships differ between contexts.

Understanding these trajectories, relationships and contrasts is especially important with respect to internal urban migration. This category accounts for the largest fraction of migration globally; over half of the world’s population resides in cities, and a vast majority of urbanites reside in low- and middle-income countries, which are experiencing the most uncontrolled urban growth and are home to 90 % of the global slum-dwelling population^(4,12)^. Those dwelling in slums, other informal urban and peri-urban settlements and the homeless disproportionately comprise voluntary urban migrants and refugees, and the destitution, infrastructural deficiencies, social exclusion and digital divides associated with these living conditions, combined with migrants’ unfamiliarity with local food and civic environments, render them less equipped to make healthy food choices^(4,13,14)^.

This study tracked changes in nutrition status, lifestyle risk factors and DoFC among migrants to two cities in Central Asia and produced an evidence base for focused research and advocacy to support locally tailored strategies for improving nutrition. The study was conducted jointly in Ulaanbaatar, the largest city in Mongolia (estimated population in 2023: 1·7 million) and Almaty, the largest city in Kazakhstan (2 million) in collaboration between the Mongolian Health Initiative and Kazakh Academy of Nutrition, using harmonised assessment methods to qualitatively compare findings across cities. These cities were considered useful comparators given a shared national heritage of nomadic pastoralism and comparable food cultures^(15–17)^, parallel transitions towards market economies status as primary migration targets in each country^(18–22)^ and dissimilar economic and migration trends in recent years. Since 2010, Mongolia has experienced extremely volatile economic growth, contributing to massive, sporadic influxes of internal migrants to Ulaanbaatar and major challenges for urban planning and infrastructure development. By contrast, Almaty has seen comparatively sustainable urbanisation due to Kazakhstan’s relatively stable economic growth, accelerating economic diversification beyond natural resources and more balanced influxes of skilled migrants.

## Methods

Participants must have migrated to Ulaanbaatar or Almaty within 2 years, intended to remain there over follow-up, not previously resided in a city and been 22–55 years old at baseline. Migrants to Ulaanbaatar were screened and randomly sampled using a database provided by the General Authority for State Registration, while Almaty migrants were sampled from sectors of the city frequently employing migrants (including vendors and maintenance workers at twenty-three public markets and those employed in cleaning and repairing public streets), adapting respondent-driven sampling methodology used in prior studies in Almaty^(23)^. Individuals were contacted by phone to introduce the study and verify eligibility using a standard script and invited to attend an informal information session led by the investigating team in each city. At each session, the team provided additional background on the study, summarised assessment procedures, addressed any questions and obtained written informed consent. The required sample size was estimated as that necessary to detect within-person changes in BMI over four repeat assessments with 80 % power at alpha = 0·05, assuming a baseline BMI of 25·8 (sd 4·0) kg/m^2^, a moderate increase over follow-up^(15)^ and a linear mixed model design; this parameterisation indicated eighty-three participants needed per city (conservatively rounded to 100)^(24)^.

A questionnaire was assembled to develop a formative understanding of how DoFC are contextualised by key domains of lifestyle risk during the process of urban migration. Assessed domains included demographics and migration history, DoFC, nutrition knowledge, dietary habits, International Physical Activity Questionnaire-Short Form, Pittsburgh Sleep Quality Index (PSQI), Generalised Anxiety Disorder 7-Item (GAD-7) Scale and Fagerström Test for Nicotine Dependence (online Supplementary Methods S1). Diet was measured in terms of frequency of consumption (< 1/week, 1/week, 2–4/week, 5–7/week, > 1/d) of twenty-three key nutritionally relevant food groups included in the Prime Diet Quality Score (PDQS), a holistic metric of diet quality designed for use in diverse populations and operationalised as a screening instrument by adapting published guidance (online Supplementary Methods S2). Reference periods over which different questions were asked varied from ‘prior to migration’ to ‘currently’ (or undefined), ‘past 2 weeks’, ‘past month’ or ‘past 3 months’; questions regarding dietary habits and nutrition knowledge were asked in reference to the past 3 months, such that the ‘combined’ reference period for these questions across the four assessments ranged 12 months, that is, from 3 months prior to migration to baseline (assessed at baseline) to 6–9 months post-migration (assessed at 9 months). Research teams at the Mongolian Health Initiative and Kazakh Academy of Nutrition evaluated the questionnaire for content validity, translated and back-translated it to and from Mongolian in Ulaanbaatar and Kazakh and Russian in Almaty, piloted it and considered iterative adjustments in coordination between teams. The final version is provided in online Supplementary Methods S3.

The questionnaire was administered in a guided fashion by research assistants, ensuring that all participants could participate regardless of literacy level, at baseline, 3, 6 and 9 months. Study nurses took clinical measurements at baseline and 9 months. Height and weight were measured by portable stadiometer and scale, waist circumference by anthropometric tape and blood pressure by automated sphygmomanometer. Accu-Check (Roche Diabetes Care, Inc.) and LipidPro (Infopia Co., Ltd) point-of-care devices were used to measure fasting blood glucose (FBG) and lipids, respectively, and body composition was analysed using TANITA SC-331S (Tanita Corporation) and Inbody 230 (InBody Co., Ltd) instruments in Ulaanbaatar and Almaty, respectively. Assessments were conducted at the Songino Khairkhan District Health Office in Ulaanbaatar and at participants’ households in Almaty. Participants received a small monetary incentive for each assessment completed.

Physical activity categories and PSQI, GAD-7 and Fagerström scores were calculated following published guidance (online Supplementary Methods S1). Dietary habits were used to calculate the PDQS, a ‘PDQS-healthy’ sub-metric and a ‘PDQS-unhealthy’ sub-metric (higher scores of which indicate higher overall diet quality, higher consumption of healthy foods and lower consumption of unhealthy foods, respectively) (online Supplementary Methods S4). A nutrition knowledge score^(25)^ (range: 0–20) was derived from responses to four questions asking whether a particular food group is generally more or less nutritious for healthy adults to consume habitually than another group and six questions asking whether it is generally more or less nutritious to consume certain food groups at all; correct, unsure and incorrect responses received two, one and zero points, respectively.

BMI was categorised using WHO global cut-offs considering evidence suggesting WHO Western Pacific Regional Office cut-offs are less applicable to Mongolian and Kazakh populations^(26,27)^. Abdominal adiposity was defined as waist circumference ≥ 102 cm (men), ≥ 88 cm (women); hypertension: systolic blood pressure ≥ 130 mmHg, diastolic blood pressure ≥ 85 mmHg or current use of antihypertensives; raised TAG: ≥ 150 mg/dl; reduced HDL-cholesterol: < 40 mg/dl (men), < 50 mg/dl (women); raised LDL-cholesterol: ≥ 160 mg/dl; normal FBG: < 6·1 mmol/l, impaired FBG: 6·1–7 mmol/l, diabetes: FBG > 7 mmol/l; and metabolic syndrome using Adult Treatment Panel III criteria (≥ 3 of the following: abdominal obesity, raised TAG, reduced HDL-cholesterol, hypertension, raised FBG)^(28)^.

Statistical analyses were conducted in R v.4.3.1 (see online Supplementary Methods S5 for packages and functions). In each city, descriptive statistics were calculated to summarise assessed characteristics at baseline and 9 months. Exploratory diet patterns were derived in each city by applying principal component analysis to food group consumption frequencies across all four assessments, and patterns were retained according to quantitative and qualitative criteria^(15)^. Mixed-effects regression models^(29)^ including a random intercept for participants were used to estimate age- and sex-adjusted associations between time since migration on measures of nutrition status, lifestyle risk and aspects of food choice and nutrition knowledge in each city. We also ran models to estimate multivariable-adjusted associations between nutrition knowledge, diet quality and diet patterns and separate models incorporating an interaction term between nutrition knowledge and migration time to evaluate whether associations changed over time. Concurrent trends in different assessed parameters were qualitatively compared within each city, and descriptive statistics, trends and associations were qualitatively compared between cities.

## Results

Two hundred participants (100 from each city) enrolled at baseline. Baseline assessments were conducted in Ulaanbaatar and Almaty in November 2019 and February 2020, respectively. Fifteen participants in Ulaanbaatar missed at least one follow-up assessment; five of these participants could be re-enrolled in subsequent assessments. Of the expected 400 person-assessments in Ulaanbaatar, 26 (6·5 %) were missed. In Almaty, six participants were lost to follow-up (two at 6 months and four at 9 months); it was not possible to re-enrol these participants. They were instead replaced with new participants at the next assessment date, such that data from 100 participants contributed to the analysis at each assessment. Demographic characteristics were comparable between the six replacement participants and the original sample (not shown).

**Figure 1.**
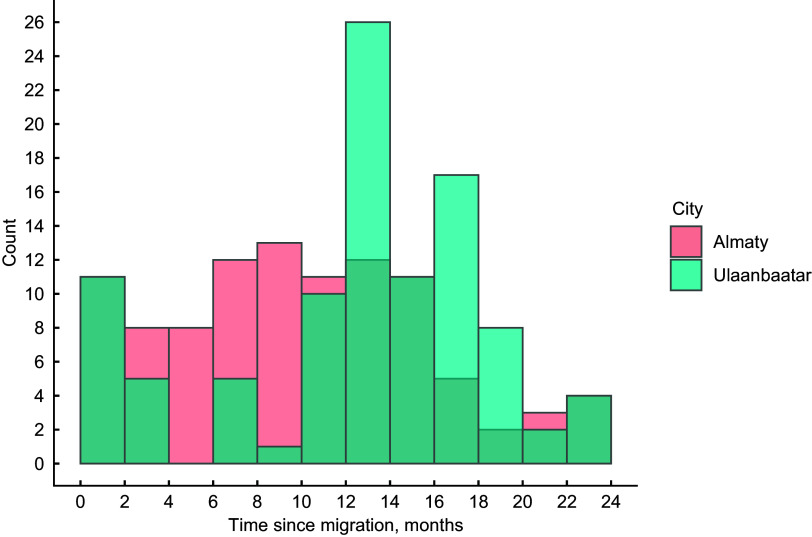
Distribution of time since migration at baseline.

In Almaty and Ulaanbaatar, respectively, 50 % and 61 % of participants were women, mean age was 33·8 (sd 9·8) and 36·6 (sd 10·5) years, 90 % were ethnic Kazakhs and Khalkha, 53 % and 77 % had at least high school education and mean time since migration was 10·4 (sd 6·0) and 13·0 (sd 5·9) months (Table 1, Fig. 1). All participants in Almaty and 52 % in Ulaanbaatar reported their primary reason for migrating was to find work; 31 % of those in Ulaanbaatar primarily migrated to join family. From prior to migration to baseline, mean household size decreased from 3·8 to 1·7 in Almaty and remained stable at 2·9 in Ulaanbaatar. In Ulaanbaatar, 46 % of participants were nomadic herders prior to migration, 55 % were unemployed at baseline, and all were employed by 9 months, while 22 % of participants in Almaty were unemployed prior to migration, none were unemployed at baseline, and the distribution of occupations remained relatively stable from baseline to 9 months.

**Table 1.** Demographic and migration characteristics assessed at baseline

Characteristic	Almaty		Ulaanbaatar	
	*n*	%	*n*	%
Age, years				
Mean	33·8		36·6	
sd	9·8		10·5	
< 30	42	42	31	31
≥ 30–< 40	27	27	28	28
≥ 40–< 50	24	24	25	25
≥ 50	7	7	16	16
Women	50	50	61	61
Ethnicity				
Kazakh or Khalkha	90	90	90	90
Other	10	10	10	10
Education level				
None or primary school	0	0	7	7
Secondary school	47	47	16	16
High school	38	38	56	56
Vocational certificate	10	10	5	5
University	4	4	15	15
Graduate or postgraduate	1	1	1	1
Time since migration, mo.				
Mean	10·4		13	
sd	6·0		5·9	
< 12	56	56	27	27
≥ 12–< 24	44	44	73	73
Migrated from				
Rural village or countryside	80	80	32	32
* Rayon* or *Soum* centre*	18	18	53	54
* Oblast* or *Aimag* centre*	2	2	14	14
Purpose of migration				
Employment	100	100	52	52
Live with family	0	0	31	31
Study	0	0	2	2
Receive medical care	0	0	2	2
Other	0	0	13	13
Household size prior to migration				
Mean	3·8		2·9	
sd	1·6		1·6	
Single person household	0	0	9	9
Household size at baseline				
Mean	1·7		2·9	
sd	1·5		1·7	
Single person household	21	21	9	9
Occupation or workplace prior to migration				
Clerk or other office job	2	2	2	2
Driver or courier	8	8	1	1
Herder or farmer	3	3	46	46
Hospital or laboratory personnel	5	5	0	0
Professional tradesperson	20	20	9	9
Restaurant or food service	2	2	4	4
Security guard	7	7	0	0
Self-employed or entrepreneur	5	5	5	5
Teacher or teaching assistant	11	11	6	6
Vendor at a shop or market	5	5	6	6
Other	11	11	2	2
Unemployed or homemaker	21	21	19	19
Occupation or workplace at baseline				
Clerk or other office job	13	13	2	2
Driver or courier	3	3	2	2
Herder or farmer	0	0	0	0
Hospital or laboratory personnel	15	15	0	0
Professional tradesperson	25	25	12	13
Restaurant or food service	6	6	6	6
Security guard	4	4	3	3
Self-employed or entrepreneur	2	2	4	4
Teacher or teaching assistant	12	12	1	1
Vendor at a shop or market	6	6	4	4
Other	14	14	5	5
Unemployed or homemaker	0	0	55	59

Given an expected sample size of 100 participants in each city at each assessment, *n* is usually equal to % for categorical variables; both statistics are presented in this and subsequent tables for consistency and to prevent confusion. *Rayon (Kazakhstan) and *Soum* (Mongolia) are district-level administrative divisions and *Oblast* (Kazakhstan) and *Aimag* (Mongolia) are province-level divisions.

**Figure 2.**
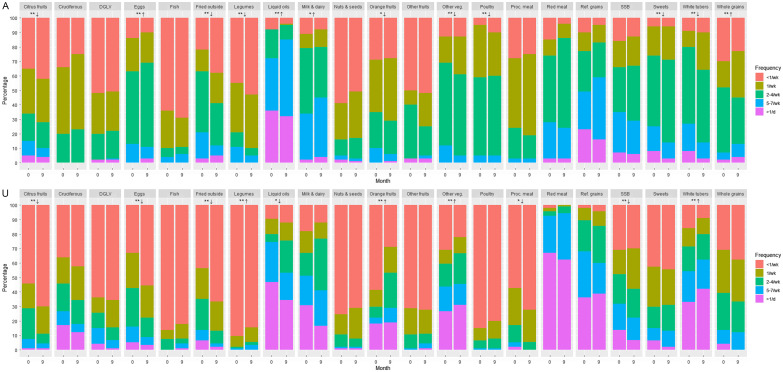
Trends in food group consumption frequencies. Panel A: Almaty; Panel U: Ulaanbaatar. Cruciferous, cruciferous vegetables; DGLV, dark green leafy vegetables; fried outside, fried foods obtained outside the home; orange fruits, deep orange fruits; proc. meat, processed meat; SSB, sugar-sweetened beverages Significance and direction of age- and sex-adjusted trends from baseline to 9 months are estimated using cumulative link mixed models and are indicated as follows: **↑, significant increase (*P* < 0·05); *↑, marginally significant increase (*P* < 0·1); **↓, significant decrease (*P* < 0·05); *↓, marginally significant decrease (*P* < 0·1); no symbols, NS (*P* > 0·10).

In Almaty, an ‘acculturated’ diet pattern accounted for 17·2 % of the variation in food group consumption and was marked by higher factor loadings for fruits, vegetables, fish, legumes, fried foods obtained outside home, whole grains and nuts and seeds (Table 2). In Ulaanbaatar, acculturated and ‘acculturating’ patterns accounted for 16·6 % and 12·0 % of variation, respectively, both were marked by higher loadings for refined grains, red meat, white tubers and milk and dairy products, and the latter also had lower factor loadings for citrus and other fruits, dark green leafy vegetables, legumes, nuts and seeds, fish, poultry, fried foods from outside home, sugar-sweetened beverages, processed meat and eggs. Migrants to Almaty increased their consumption of eggs, whole grains, liquid oils (*P* < 0·05) and milk and dairy products (*P* < 0·1) and decreased that of poultry, fried foods from outside home, white tubers, sweets, citrus fruits, legumes, other vegetables (*P* < 0·05) and deep orange fruits (*P* < 0·1), while migrants to Ulaanbaatar increased the consumption of white tubers, deep orange fruits, legumes and other vegetables (*P* < 0·05) and decreased that of eggs, fried foods from outside home, sugar-sweetened beverages, citrus fruits (*P* < 0·05), processed meat and liquid oils (*P* < 0·05) over follow-up (Fig. 2).

**Table 2.**
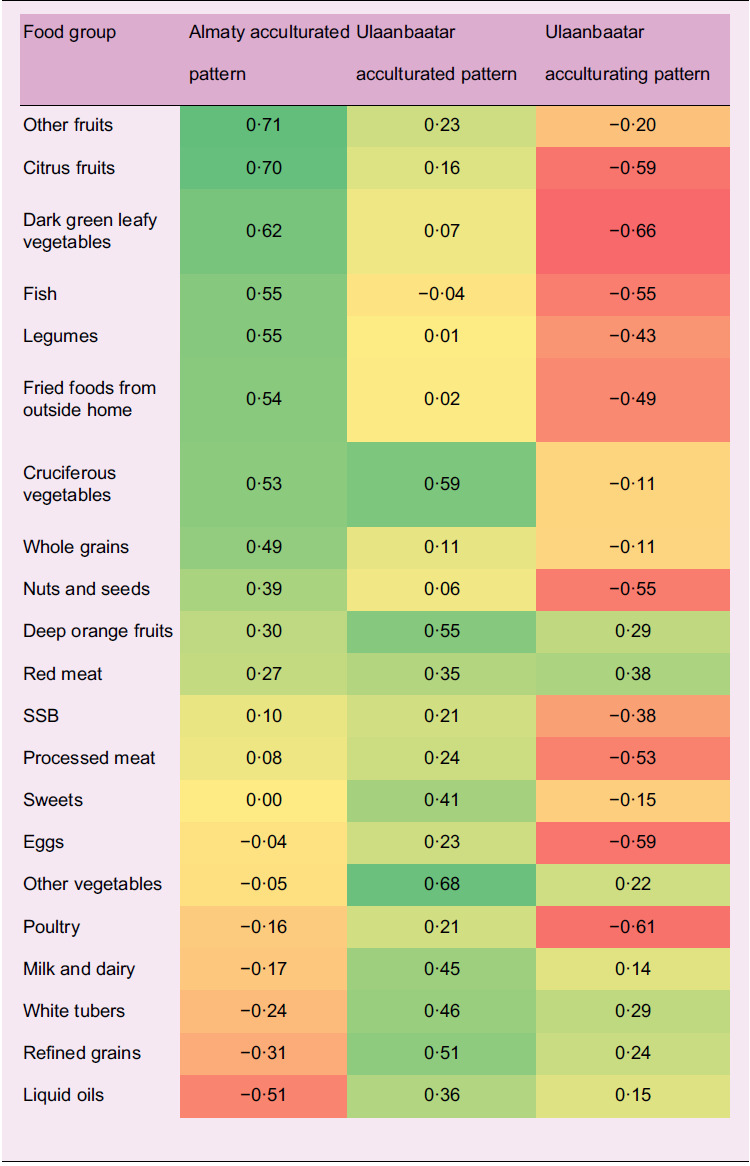
Exploratory diet pattern factor loadings

SSB, sugar-sweetened beverages. The acculturated diet pattern accounted for 17·2 % of the variation in food group consumption in Almaty and the acculturated and acculturating patterns accounted for 16·6 % and 12·0 % of variation in Ulaanbaatar, respectively. Shading is proportional to the magnitude and direction of factor loadings (darkest green: 0·71, yellow: 0 %, darkest red: –0·66).

At baseline, in Almaty and Ulaanbaatar, respectively, 43 % and 47 % of participants were abdominally obese, 6 % and 22 % were hypertensive, 3 % and 18 % were prediabetic or diabetic, 35 % and 33 % had reduced HDL-cholesterol, 3 % and 1 % had raised LDL-cholesterol, 5 % and 42 % had raised TAG and 3 % and 22 % had metabolic syndrome (Table 3). Each month since migration to Almaty was associated with increased fasting TAG (95 % CI: 0·13, 0·94; *P* = 0·007), while each month since migration to Ulaanbaatar was associated with increased BMI (*β* = 0·04 kg/m^3^; 95 % CI: 0·01, 0·07; *P* = 0·023), body fat percentage (*β* = 0·14 %; 95 % CI: 0·01, 0·26; *P* = 0·032), FBG (*β* = 0·04 mmol/l; 95 % CI: 0·02, 0·05; *P* < 0·0001), total cholesterol (*β* = 0·58 mg/dl, 95 % CI: 0·02, 1·12; *P* = 0·042), LDL-cholesterol (*β* = 0·54 mg/dl, 95 % CI: 0·12, 0·95; *P* = 0·012) and HDL-cholesterol (*β* = 0·26 mg/dl; 95 % CI: 0·03, 0·50; *P* = 0·026). A marginally significant increase in waist circumference was also observed with each month since migration to Ulaanbaatar (*β* = 0·09 cm; 95 % CI: –0·01, 0·19; *P* = 0·090).

**Table 3.** Trends in anthropometric and clinical measurements

	Almaty							Ulaanbaatar						
Measurement	Baseline		9 months					Baseline		9 months		*β* ^†^	95 % CI	*P* ^†^
	Mean	sd	Mean	sd	*β* ^†^	95 % CI	*P* ^†^	Mean	sd	Mean	sd			
BMI, kg/m^3	24·4	4·6	24·5	4·5	0·00	–0·03, 0·02	0·935	25·9	4·7	26·5	5·0	0·04	0·01, 0·07	0·023**
	*n*	%	*n*	%				*n*	%	*n*	%			
< 18·5	7	7	5	5				2	2	1	1			
≥ 18–< 25	53	53	57	57				45	45	39	43			
≥ 25–< 30	27	27	26	26				36	36	31	34			
≥ 30	13	13	12	12				16	16	19	21			
	Mean	sd	Mean	sd				Mean	sd	Mean	sd			
Waist circumference, cm	83·3	14·3	83·0	13·6	−0·04	–0·12, 0·04	0·379	83·5	10·7	84·1	10·7	0·09	–0·01, 0·19	0·090*
Among men only	90·7	12·2	89·6	11·8				82·5	10·4	83·8	10·9			
Among women only	75·9	12·3	76·3	12·0				84·1	10·9	84·3	10·7			
	*n*	%	*n*	%				*n*	%	*n*	%			
Normal	57	57	59	59				53	53	45	50			
Abdominal obesity	43	43	41	41				47	47	45	50			
	Mean	sd	Mean	sd				Mean	sd	Mean	sd			
Body fat percentage	28·7	14·6	28·7	14·0	0·00	–0·26, 0·26	0·988	27·6	10·7	29·3	13·0	0·14	0·01, 0·26	0·032**
Among men only	24·8	8·5	27·2	17·9				19·3	6·5	20·4	7·8			
Among women only	32·5	18·2	30·3	8·2				33·0	9·4	34·5	12·7			
Systolic BP, mmHg	117·6	14·0	118·2	12·9	0·03	–0·09, 0·14	0·636	124·9	22·2	122·6	17·1	−0·17	–0·44, 0·11	0·215
Diastolic BP, mmHg	75·1	8·5	74·8	7·5	−0·05	–0·13, 0·04	0·293	82·5	15·7	83·3	12·9	0·02	–0·17, 0·22	0·815
	*n*	%	*n*	%				*n*	%	*n*	%			
Hypertension	6	6	9	9				22	22	17	19			
	Mean	sd	Mean	sd				Mean	sd	Mean	sd			
Blood glucose, mmol/l^‡^	4·8	0·7	4·7	0·7	0·00	–0·01, 0·01	0·563	5·4	0·9	5·9	0·9	0·04	0·02, 0·05	< 0·001**
	*n*	%	*n*	%				*n*	%	*n*	%			
Normal	97	97	97	97				80	80	53	60			
Impaired	0	0	1	1				12	12	22	25			
Diabetic	3	3	2	2				8	8	14	16			
	Mean	sd	Mean	sd				Mean	sd	Mean	sd			
Total cholesterol, mg/dl^‡^	173·0	31·3	171·3	24·5	−0·15	–0·52, 0·23	0·443	160·1	38·7	168·9	39·5	0·58	0·02, 1·12	0·042**
LDL-cholesterol, mg/dl^‡^	102·2	33·0	95·7	29·2	−0·29	–0·71, 0·12	0·166	78·2	30·2	85·4	30·2	0·54	0·12, 0·95	0·012**
	*n*	%	*n*	%				*n*	%	*n*	%			
Normal	97	97	98	98				95	99	79	98			
Raised	3	3	2	2				1	1	2	2			
	Mean	sd	Mean	sd				Mean	sd	Mean	sd			
HDL-cholesterol, mg/dl^‡^	53·9	14·1	56·8	16·2	0·02	–0·08, 0·12	0·711	51·8	13·9	54·9	15·0	0·26	0·03, 0·5	0·026**
Among men only	50·9	12·8	55·2	17·0				48·1	12·6	50·8	13·3			
Among women only	57·0	14·7	58·4	15·3				54·0	14·2	57·2	14·5			
	*n*	%	*n*	%				*n*	%	*n*	%			
Normal	65	65	68	68				66	66	61	61			
Reduced	35	35	32	32				32	32	28	28			
	Mean	sd	Mean	sd				Mean	sd	Mean	sd			
TAG, mg/dl^‡^	84·3	29·2	94·0	25·8	0·55	0·13, 0·94	0·007**	142·6	80·0	141·3	86·6	−0·63	–1·86, 0·62	0·322
	*n*	%	*n*	%				*n*	%	*n*	%			
Normal	95	95	97	97				57	58	59	66			
Raised	5	5	3	3				42	42	31	34			
	Mean	sd	Mean	sd				Mean	sd	Mean	sd			
MetS components, #	0·9	0·9	0·9	0·9	0·00	–0·01, 0·01	0·689	1·6	1·2	1·8	1·3	0·00	–0·02, 0·02	0·732
	*n*	%	*n*	%				*n*	%	*n*	%			
< 3 (MetS absent)	97	97	94	94				76	78	64	73			
≥ 3 (MetS present)	3	3	6	6				22	22	24	27			

BP, blood pressure; MetS, metabolic syndrome. ^†^ *β* (95 % CI) and *P* statistics indicate the age- and sex-adjusted parameter estimate and *P* value for the association between a 1-month increase in time since migration and each continuous outcome estimated using linear mixed models (these statistics are omitted for binary and ordered categorical outcomes). ^‡^Glucose and lipids were measured in fasting samples. *P < 0·05, **P < 0·01.

In Almaty and Ulaanbaatar, respectively, 31 % and 38 % of participants smoked and 23 % and 63 % of smokers were moderately nicotine dependent, 19 % and 32 % of participants had low physical activity, 27 % and 68 % had disturbed sleep and 2 % and 9 % had at least moderate generalised anxiety at baseline (Table 4). Each month since migration to Almaty was associated with increased PDQS-unhealthy sub-metric scores (*β* = 0·05; 95 % CI: 0·00, 0·09; *P* = 0·032) indicating lower consumption of unhealthy foods, PSQI scores (*β* = 0·02; 95 % CI: 0·00, 0·05; *P* = 0·042) indicating declining sleep quality and marginally significantly increased GAD-7 scores (*β* = 0·04; 95 % CI: –0·00, 0·09; *P* = 0·063) indicating worsening anxiety. Among migrants to Ulaanbaatar, each month since migration was associated with decreased PSQI (*β* = –0·09; 95 % CI: –0·13, –0·04; *P* < 0·001) and GAD-7 scores (*β* = –0·12; 95 % CI: –0·16, –0·06; *P* < 0·001) and increased odds of smoking (*β* = 1·26; 95 % CI: 1·01, 1·57; *P* = 0·039) albeit decreased Fagerström scores (*β* = –0·04; 95 % CI: –0·07, –0·01; *P* = 0·008) indicating less physical addiction. Each month since migration to Ulaanbaatar was also marginally significantly associated with increased PDQS scores (*β* = 0·07; 95 % CI: –0·01, 0·16; *P* = 0·096) and adherence to the acculturating diet pattern (*β* = 0·28; 95 % CI: –0·03, 0·58; *P* = 0·074).

**Table 4.** Trends in lifestyle risk factors for chronic disease

	Almaty							Ulaanbaatar						
Risk factor	Baseline		9 months		*β* or OR^†^	95 % CI	*P* ^†^	Baseline		9 months		*β* or OR^†^	95 % CI	*P* ^†^
	Mean	sd	Mean	sd				Mean	sd	Mean	sd			
PDQS score (range: 0–80)	31·1	4·9	31·5	5·3	0·04	–0·03, 0·11	0·263	27·7	5·4	27·9	5·6	0·07	–0·01, 0·16	0·096*
PDQS-healthy score (range: 0–52)	16·0	5·0	15·7	4·7	0·00	–0·07, 0·07	0·910	13·1	6·5	12·3	5·8	0·03	–0·06, 0·12	0·541
PDQS-unhealthy score (range: 0–28)	15·1	2·8	15·8	2·7	0·05	0·00, 0·09	0·032**	14·6	4·0	15·6	3·5	0·04	–0·01, 0·10	0·141
Acculturated diet pattern (scaled from 0–100)	37·6	20·8	35·1	20·5	−0·16	–0·47, 0·15	0·302	47·2	20·9	45·3	19·0	0·12	–0·18, 0·43	0·430
Acculturating diet pattern (scaled from 0–100)	NA		NA		NA		NA	66·2	21·4	72·4	18·9	0·28	–0·03, 0·58	0·074*
Self-described diet quality					0·02	–0·06, 0·10	0·615					−0·01	–0·06, 0·04	0·800
	*n*	%	*n*	%				*n*	%	*n*	%			
Very healthy	1	1	2	2				10	10	7	8			
Healthy	35	35	29	29				32	33	25	28			
Average	58	58	58	58				49	50	53	59			
Unhealthy	5	5	10	10				7	7	4	4			
Very unhealthy	1	1	1	1				0	0	1	1			
	Mean	sd	Mean	sd				Mean	sd	Mean	sd			
Nutrition knowledge score (range: 0–20)	13·0	4·2	11·1	4·7	−0·15	–0·21, –0·08	< 0·001**	12·7	2·7	12·9	2·8	0·02	–0·02, 0·06	0·256
IPAQ physical activity category					−0·04	–0·12, 0·04	0·296					0·00	–0·05, 0·05	0·943
	*n*	%	*n*	%				*n*	%	*n*	%			
High	59	59	44	44				54	54	49	54			
Medium	22	22	33	33				14	14	12	13			
Low	19	19	23	23				32	32	29	32			
Self-described physical activity					0·04	–0·05, 0·14	0·378					0·04	–0·01, 0·09	0·107
High	5	5	12	12				25	28	19	21			
Moderate	84	84	71	71				51	57	45	50			
Low	11	11	17	17				23	26	26	29			
	Mean	sd	Mean	sd				Mean	sd	Mean	sd			
Sedentary time, h/d	3·8	3·2	3·8	1·9	−0·04	–0·12, 0·03	0·294	3·6	3·0	3·4	2·8	0·00	–0·05, 0·04	0·867
PSQI score (range: 0–21)	3·7	1·7	4·0	1·4	0·02	0·00, 0·05	0·042**	6·1	3·1	5·0	3·2	−0·09	–0·13, –0·04	< 0·001**
	*n*	%	*n*	%				*n*	%	*n*	%			
Normal sleep quality	73	73	57	57				32	32	52	52			
Disturbed sleep	27	27	43	43				68	68	48	48			
Self-described sleep quality					0·04	–0·01, 0·1	0·126					−0·08	–0·13, –0·03	0·003**
Very good	36	36	24	24				15	15	23	26			
Fairly good	59	59	73	73				60	60	45	50			
Fairly poor	5	5	3	3				21	21	20	22			
Very poor	0	0	0	0				4	4	2	2			
Current smoker	31	31	31	31	0·03	–0·23, 0·3	0·831	38	38	39	39	0·23	0·01, 0·45	0·039**
	Mean	sd	Mean	sd				Mean	sd	Mean	sd			
Fagerström score (range: 0–10)	3·0	1·8	3·0	1·8	−0·01	–0·02, 0·00	0·214	4·7	1·0	3·6	1·5	−0·04	–0·07, –0·01	0·008**
	*n*	%	*n*	%				*n*	%	*n*	%			
Low nicotine dependence	15	48	15	48				1	3	10	26			
Low to moderate nicotine dependence	9	29	10	32				13	42	19	49			
Moderate nicotine dependence	7	23	6	19				24	77	10	26			
High nicotine dependence	0	0	0	0				0	0	0	0			
	Mean	sd	Mean	sd				Mean	sd	Mean	sd			
GAD-7 score (range: 0–21)	2·0	2·3	2·8	2·3	0·04	0·00, 0·09	0·063*	4·9	3·8	2·3	4·0	−0·11	–0·16, –0·06	< 0·001**
	*n*	%	*n*	%				*n*	%	*n*	%			
Minimal generalised anxiety	94	94	79	79				47	47	57	63			
Mild generalised anxiety	4	4	20	20				43	43	25	28			
Moderate generalised anxiety	2	2	1	1				7	7	7	8			
Severe generalised anxiety	0	0	0	0				2	2	1	1			
Self-described bodyweight					0·15	0·01, 0·29	0·042**					0·08	–0·06, 0·25	0·283
Overweight	20	20	28	28				37	41	36	40			
Normal	79	79	65	65				53	59	47	52			
Underweight	1	1	7	7				9	10	7	8			

PDQS, Prime Diet Quality Score; IPAQ, International Physical Activity Questionnaire; PSQI, Pittsburgh Sleep Quality Index; GAD, Generalised Anxiety Disorder; NA, not applicable. ^†^ *β* or OR (95 % CI) and *P* statistics indicate the age- and sex-adjusted parameter estimate (for continuous outcomes) or OR (for binary and ordered categorical outcomes) and *P* value for the association between a 1-month increase in time since migration and each outcome, estimated using linear mixed models for continuous outcomes, generalised linear mixed models for one binary outcome (PSQI category) and cumulative link mixed models for ordered categorical outcomes (OR for binary and ordered categorical outcomes is that associated with a one-row descent in ordered category presented in the table, e.g. the OR for ‘IPAQ physical activity category’ is that associated with being in either the ‘Moderate’ *v*. ‘High’ category or ‘Low’ *v*. ‘Moderate’ category). *P < 0·05, **P < 0·01.

Each month since migration to Almaty was associated with increased influence of local food availability (OR for one-unit change in ordered category = 1·20; 95 % CI: 1·12, 1·30; *P* < 0·001), price (OR = 1·19; 95 % CI: 1·11, 1·27; *P* < 0·001) and taste (OR = 1·04; 95 % CI: 1·03, 1·04; *P* < 0·001) on food choices and marginally significant increases in the influence of the time, effort or skill required to cook foods (OR = 1·07; 95 % CI: 1·00, 1·15; *P* = 0·055) and their nutritive quality (OR = 1·01; 95 % CI: 0·94, 1·08; *P* = 0·076) (Table 5). By contrast, each month since migration to Ulaanbaatar was associated only with a marginally significant decrease in the influence of local availability of foods (OR = 0·96; 95 % CI: 0·92, 1·00; *P* = 0·068). With each month since migration, migrants were more likely to report that healthy foods were less available than healthy ones in Almaty (OR = 0·97; 95 % CI: 0·97, 0·97; *P* < 0·001) and that healthy foods were easier to cook than unhealthy ones in Ulaanbaatar (OR = 1·05; 95 % CI: 1·00, 1·10; *P* = 0·056). Non-significant trends (*P* > 0·10) are presented in online Supplementary Results.

**Table 5.** Statistically significant (*P* < 0·05) trends in drivers of food choice and related perceptions and behaviours

		Almaty							Ulaanbaatar						
Question	Response	Baseline		9 months		OR^†^	95 % CI	*P* ^†^	Baseline		9 months		OR^†^	95 % CI	*P* ^†^
		*n*	%	*n*	%				*n*	%	*n*	%			
Influence of the availability of food where you live on your food choice	None	0	0	0	0	1·20	1·12, 1·30	< 0·001**	21	21	18	20	0·96	0·92, 1·00	0·068*
	Weak	22	22	7	7				22	22	19	21			
	Moderate	47	47	26	26				22	22	30	33			
	Strong	31	31	62	62				22	22	15	17			
	Very strong	0	0	5	5				13	13	8	9			
Influence of the price of food on your food choices	None	15	15	3	3	1·19	1·11, 1·27	< 0·001**	17	17	12	13	1·01	0·96, 1·06	0·658
	Weak	0	0	0	0				18	18	16	18			
	Moderate	51	51	28	28				31	31	24	27			
	Strong	31	31	63	63				22	22	26	29			
	Very strong	3	3	6	6				12	12	12	13			
Influence of the taste of food on your food choice	None	0	0	0	0	1·04	1·03, 1·04	< 0·001**	17	17	17	19	0·98	0·94, 1·03	0·484
	Weak	18	18	4	4				18	18	13	14			
	Moderate	29	29	46	46				24	24	31	34			
	Strong	53	53	49	49				28	28	24	27			
	Very strong	0	0	1	1				13	13	5	6			
Influence of the nutritive quality of food on your food choice	None	12	12	7	7	1·01	0·94, 1·08	0·076*	8	8	11	12	0·99	0·95, 1·04	0·692
	Weak	15	15	14	14				17	17	10	11			
	Moderate	58	58	61	61				33	33	34	38			
	Strong	15	15	18	18				30	30	26	29			
	Very strong	0	0	0	0				12	12	9	10			
Influence of the time, effort or skill required to cook food on your food choice	None	12	12	7	7	1·07	1, 1·15	0·055*	24	24	26	29	1·00	0·95, 1·04	0·875
	Weak	6	6	6	6				20	20	13	14			
	Moderate	64	64	54	54				23	23	25	28			
	Strong	16	16	32	32				19	19	13	14			
	Very strong	2	2	1	1				14	14	13	14			
Compared with unhealthy foods, healthy foods are generally…	Much less available	7	7	14	14	0·97	0·97, 0·97	< 0·001**	5	5	3	3	1·02	0·97, 1·08	0·355
	Less available	21	21	14	14				22	22	15	17			
	Equally available	66	66	67	67				34	34	39	43			
	More available	6	6	5	5				36	36	27	30			
	Much more available	0	0	0	0				3	3	6	7			
Compared with unhealthy foods, healthy foods are generally…	Much harder to cook	2	2	2	2	1·01	0·95, 1·09	0·691	4	4	1	1	1·05	1·00, 1·10	0·056*
	Harder to cook	17	17	16	16				10	10	6	7			
	Same difficulty	40	40	33	33				45	45	43	48			
	Easier to cook	30	30	40	40				38	38	37	41			
	Much easier to cook	11	11	9	9				2	2	3	3			
‘My diet influences my bodyweight’.	Strongly disagree	1	1	0	0	1·03	1·02, 1·03	< 0·001**	8	8	7	8	0·98	0·93, 1·03	0·504
	Disagree	9	9	6	6				9	9	8	9			
	Neutral	21	21	27	27				16	16	15	17			
	Agree	61	61	47	47				44	44	44	49			
	Strongly agree	8	8	20	20				23	23	16	18			
‘I pay attention to nutrition information on food packaging’.	Strongly disagree	9	9	2	2	1·04	1·04, 1·04	< 0·001**	6	6	4	4	1·01	0·96, 1·06	0·752
	Disagree	19	19	16	16				11	11	8	9			
	Neutral	25	25	30	30				12	12	17	19			
	Agree	42	42	44	44				49	49	40	44			
	Strongly agree	5	5	8	8				22	22	21	23			
‘I eat worse when I am stressed, depressed or tired’.	Strongly disagree	24	24	15	15	1·08	1·02, 1·14	0·004**	19	19	10	11	0·99	0·94, 1·04	0·696
	Disagree	15	15	11	11				21	21	26	29			
	Neutral	33	33	28	28				16	16	14	16			
	Agree	24	24	35	35				34	34	29	32			
	Strongly agree	4	4	11	11				10	10	11	12			
How would you characterise your own cooking skills?	Very skilled	5	5	5	5	1·07	1·07, 1·07	< 0·001**	10	10	7	8	1·02	0·96, 1·08	0·552
	Skilled	30	30	24	24				44	44	40	44			
	Average	53	53	55	55				45	45	41	46			
	Poor	10	10	15	15				0	0	2	2			
	Very poor	2	2	1	1				0	0	0	0			
How frequently has your household cooked its meals?	All meals	11	11	23	23	0·88	0·81, 0·95	0·002**	65	66	52	58	1·02	0·95, 1·08	0·635
	Most meals	52	52	44	44				33	33	33	37			
	Some meals	35	35	28	28				0	0	1	1			
	Few meals	1	1	3	3				1	1	3	3			
	No meals	1	1	2	2				0	0	1	1			
How often has your household eaten together? (multi-person households only)	All meals	36	36	44	44	0·89	0·82, 0·98	0·016**	60	67	50	60	1·05	0·99, 1·11	0·143
	Most meals	35	35	25	25				19	21	22	27			
	Some meals	5	5	9	9				8	9	10	12			
	Few meals	3	3	0	0				1	1	1	1			
	No meals	0	0	1	1				2	2	0	0			

^†^OR (95 % CI) and *P* statistics indicate the age- and sex-adjusted OR and *P* value for the association between a 1-month increase in time since migration and each outcome, estimated using cumulative link mixed models (OR is that associated with a one-row descent in ordered category presented in the table, e.g. the OR for ‘Influence of the price of food on your food choices’ is that associated with being in either the ‘Weak’ *v*. ‘None’ category, ‘Moderate’ *v*. ‘Weak’ category, ‘Strong’ *v*. ‘Moderate’ category or ‘Very strong’ *v*. ‘Strong’ category). *P < 0·05, **P < 0·01.

**Figure 3.**
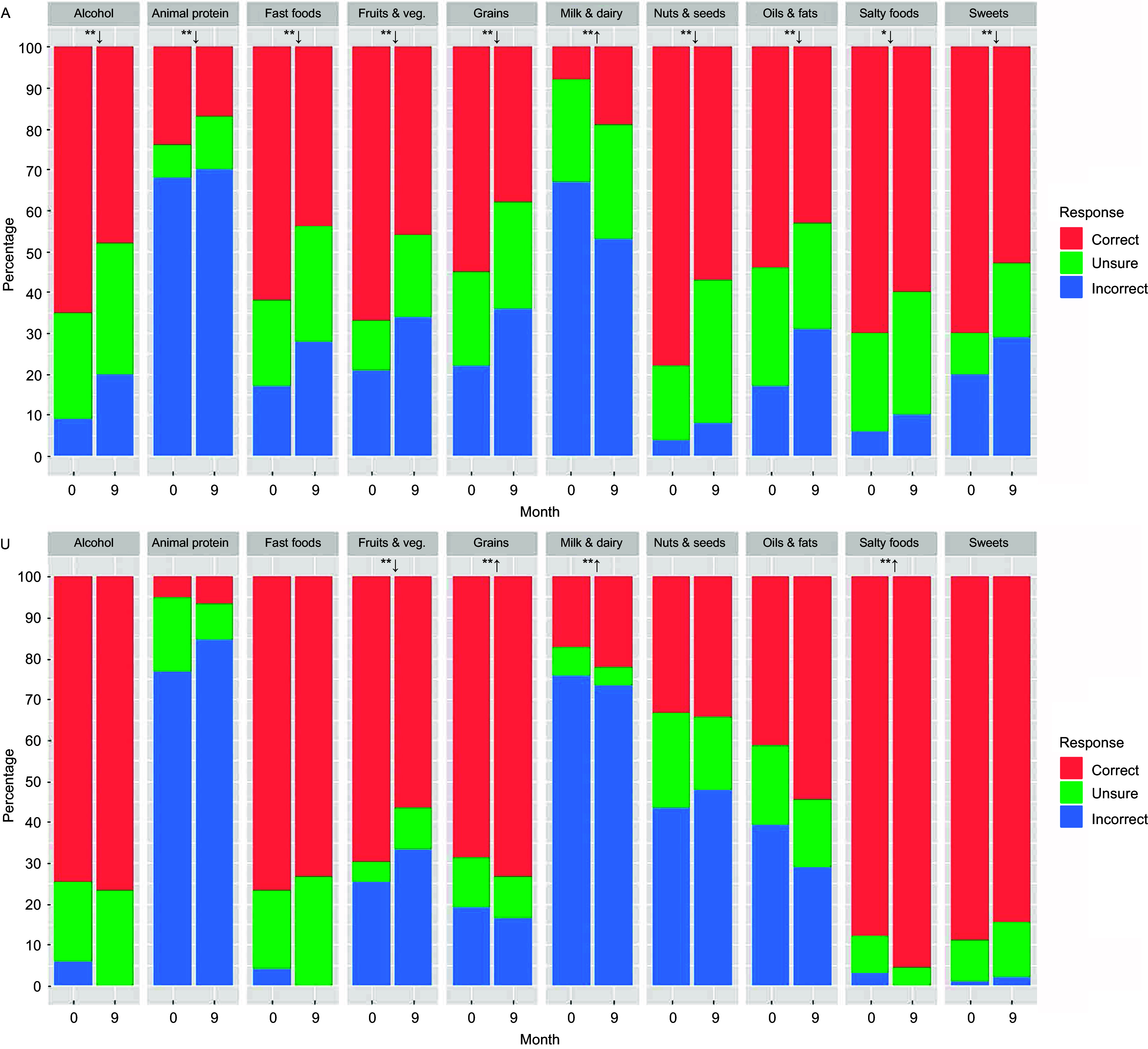
Trends in nutrition knowledge components. Panel A: Almaty; Panel U: Ulaanbaatar. Bar heights indicate the proportion of correct, unsure and incorrect responses to four questions asking whether it is generally more nutritious for healthy adults to habitually consume either (1) ‘red meat *v*. lean meat (e.g. chicken, fish)’ (abbreviated as ‘Animal protein’ in the figure), (2) ‘whole fat *v*. reduced fat milk and dairy products’ (‘Milk & dairy’), (3) ‘liquid oils *v*. animal fats’ (‘Oils & fats’) and (4) ‘whole *v*. refined grains and grain products’ (‘Grains’) and six questions asking whether it is generally more nutritious for healthy adults to habitually consume more or less of (5) ‘salt and salty foods’ (‘Salty foods’), (6) ‘sugar and sugary foods and drinks’ (‘Sweets’), (7) ‘fruits and vegetables’ (‘Fruits & veg.’), (8) ‘nuts and seeds’ (‘Nuts & seeds’), (9) ‘processed and fast foods’ (‘Fast foods’) and (10) ‘alcoholic drinks’ (‘Alcohol’). Significance and direction of age- and sex-adjusted trends from baseline to 9 months are estimated using cumulative link mixed models and are indicated as follows: **↑, significant increase (*P* < 0·05); **↓, significant decrease (*P* < 0·05); *↓, marginally significant decrease (*P* < 0·1); no symbols, NS (*P* > 0·10).

Each month since migration was associated with decreased nutrition knowledge scores in Almaty (*β* = –0·15; 95 % CI: –0·21, –0·08; *P* < 0·001) but not Ulaanbaatar (Table 4). The proportion of correct responses to eight of ten nutrition knowledge questions among migrants to Almaty decreased significantly (*P* < 0·05), while a marginally significant decrease (*P* < 0·1) and significant increase (*P* < 0·05) were observed for questions about salty foods and high *v*. low fat dairy, respectively (Fig. 3). By contrast, the proportion of correct responses among migrants to Ulaanbaatar increased for questions on high *v*. low fat dairy, whole *v*. refined grains and salty foods (*P* < 0·05); decreased for the question on fruits and vegetables (*P* < 0·05); and were otherwise non-significant (*P* > 0·10).

Adjusting for age, sex, migration time, education, ethnicity, physical activity, smoking and household type (single- *v*. multi-person), nutrition knowledge scores were positively associated with PDQS (*β* = 0·37; 95 % CI: 0·27, 0·47; *P* < 0·001) and PDQS-unhealthy scores (*β* = 0·11; 95 % CI: 0·05, 0·17; *P* < 0·001) among migrants to Almaty and marginally associated with higher PDQS-healthy (*β* = 0·21; 95 % CI: –0·02, 0·46; *P* = 0·087) and PDQS-unhealthy scores among migrants to Ulaanbaatar (*β* = 0·05; 95 % CI: –0·01, 0·10; *P* = 0·092) (Table 6). Nutrition knowledge scores were also positively associated with adherence to acculturated diet patterns in both Almaty (*β* = 0·56; 95 % CI: 0·12, 1·02; *P* = 0·016) and Ulaanbaatar (*β* = 1·43; 95 % CI: 0·64, 2·23; *P* < 0·001). Among migrants to Almaty, time since migration modified the association between nutrition knowledge and the PDQS-unhealthy sub-metric, such that for each month since migration, the strength of this association decreased by 0·01 points (95 % CI: 0·00, 0·02; *P*-interaction = 0·008).

**Table 6.** Associations between nutrition knowledge, diet quality and diet patterns

	Almaty						Ulaanbaatar					
	Main effect^†^			Interaction term^‡^			Main effect^†^			Interaction term^‡^		
Outcome	*β*	95 % CI	*P*	*β*	95 % CI	*P*	*β*	95 % CI	*P*	*β*	95 % CI	*P*
PDQS score (range: 0–80)	0·37	0·27, 0·47	< 0·001**	−0·01	–0·02, 0·00	0·171	−0·08	–0·31, 0·15	0·497	0·00	–0·02, 0·03	0·747
PDQS-healthy score (range: 0–52)	0·03	0·15, 0·35	0·365	0·00	–0·01, 0·01	0·706	0·21	–0·02, 0·46	0·087*	−0·01	–0·04, 0·02	0·696
PDQS-unhealthy score (range: 0–28)	0·11	0·05, 0·17	< 0·001**	−0·01	–0·02, 0·00	0·008**	0·05	–0·01, 0·10	0·092*	0·01	–0·01, 0·03	0·284
Acculturated diet pattern (scaled from 0 to 100)	0·56	0·12, 1·02	0·016**	0·04	–0·01, 0·09	0·121	1·43	0·64, 2·23	0·001**	−0·09	–0·19, 0·02	0·109
Acculturating diet pattern (scaled from 0 to 100)	NA		NA	NA		NA	−0·12	–0·94, 0·69	0·780	−0·10	–0·20, 0·00	0·057*

PDQS, Prime Diet Quality Score; NA, not applicable. ^†^ *β* (95 % CI) and *P* statistics for main effects indicate the age- and sex-adjusted parameter estimate and *P* value for the association between a one-unit increase in nutrition knowledge score (range: 0–20) and each outcome. ^‡^ *β* (95 % CI) and *P* statistics for interaction terms are estimated using a separate set of models incorporating an interaction term between nutrition knowledge score and time since migration (in months). Models are estimated using linear mixed-effects models adjusted for age, sex, time since migration, education level, ethnicity, physical activity category, smoking and household type (single *v*. multi-person). *P < 0·05, **P < 0·01.

## Discussion

In analysis of panel data on first-time migrants to Ulaanbaatar and Almaty, migrants to Ulaanbaatar had a moderate prevalence of metabolic and lifestyle risk factors for chronic disease at baseline and incurred deteriorations in metabolic indicators over follow-up. These findings generally agree with studies across diverse low- and middle-income countries that tend to observe cardiometabolically deleterious shifts following urban migration, including increasing gradients in weight-for-height across rural, urban migrant and urban host populations^(30,31)^ that we also found in prior investigations in Mongolia^(15,16)^. However, the relationship between migration and nutrition is heterogeneous (in the present study, metabolic and lifestyle health among migrants to Almaty were comparatively good at baseline and changed little over time), and despite risks, urban migration can improve access to fruits and vegetables^(16,32,33)^ and be a potentially advantageous adaption strategy for household livelihoods^(34–36)^.

Acculturating and acculturated diet patterns among migrants to Ulaanbaatar share similarities in factor loadings with ‘nomadic’ and ‘urban’ patterns, respectively, which we previously identified in a nationwide survey of Mongolians^(15)^. In that survey, rural nomadic and urban host populations adhered more strongly to the nomadic and urban patterns, respectively, and adherence to the urban (but not nomadic) pattern was associated with higher BMI after adjustment for total energy intake. Collectively, these findings suggest the process of assimilating urban food culture in Mongolia – marked by transitions from nomadic to acculturating, acculturated and urban diet patterns – may have contributed to observed deteriorations in metabolic health among migrants to Ulaanbaatar. In our prior survey, we also found rural Mongolians adhered more to the nomadic dietary pattern in summer than in winter^(15)^. Seasonal changes in food availability may explain the marginally significant increase in adherence to the nomadic-like acculturating diet pattern (but not the acculturated one) observed among migrants to Ulaanbaatar over follow-up from November (start of winter in Mongolia) to August (end of summer). These findings, and the fact that all four patterns identified in the prior and current studies share positive factor loadings for two food groups comprising 60 % of the national diet by mass (red meat and refined grains)^(15)^, suggest nomadic transitions remain strongly influential on the diet of urban migrants throughout acculturation. Increased use of traditional foods by migrants during early acculturation may also reflect a greater degree of choice, nostalgia or neophobia linked to acculturative stress following initial familiarisation with new environments^(37,38)^.

In comparison with migrants to Almaty, DoFC and related perceptions and behaviours were largely uninfluenced by migration to Ulaanbaatar. This may be explained by differences in household migration patterns between Mongolia and Kazakhstan. Internal migration in Mongolia is typified as a sequence of movements from the countryside to tertiary, district and provincial centres and finally, Ulaanbaatar^(19)^, during which households increasingly acculturate to urban lifestyles. As observed in studies of international migration, kinship is an important aspect of the migration process in Mongolia, families are a key source of information in migration decisions, and family members typically migrate together even if only one has secured employment at the destination^(18,19)^, factors which buffer migration-related shocks. However, despite their relative assimilation of urban food culture, urban migrants in Mongolia usually reside in peri-urban slums^(39)^ where deprivation of the food environment challenges migrants’ access to healthy foods^(4)^. By contrast, while migrants to Kazakh cities usually find permanent housing there^(40)^, internal migration in Kazakhstan is costly in comparison with other countries, and finding employment is a major priority^(21,22)^. Resultingly, urban migrants in Kazakhstan frequently move individually and directly from the periphery instead of in a stepwise fashion, both characteristics which we observed in the present study. The comparatively abrupt and often solitary nature of internal migration in Kazakhstan may have a disruptive effect on intra- and intergenerational understanding of healthy foods and unhealthy foods, may render migrants more susceptible to internalising persuasive marketing tactics and misleading information disseminated by fast and processed food corporations and may have contributed to observed declines in nutrition knowledge^(4–7,13,14)^.

A recent study among urban poor in Vietnam found an objective nutrition knowledge scale was associated with higher consumption of healthy dietary components and lower consumption of starchy staples and sodium^(41)^, and studies in other countries have found education of household heads is positively correlated with diet quality^(42)^. Among migrants to Almaty, we observed significant and positive, multivariable-adjusted associations between objective nutrition knowledge and diet quality. It is also possible that among environmental and infrastructural changes associated with urban migration to Almaty (including upgraded living standards), shifts in perceptions and behaviours related to food choice and at least somewhat unrelated to nutrition knowledge – for example, influence of price, taste and availability on food choice decisions and cooking skills, use of food packaging and perceived influence of diet on bodyweight – partially replaced nutrition knowledge as determinants of diet quality. This hypothesis is based on three circumstantial observations: Almaty migrants reported concurrent increases in all the aforementioned perceptions and behaviours (and others); unhealthy food consumption improved despite a concurrent decline in nutrition knowledge; and the multivariable-adjusted association between these improvements and nutrition knowledge attenuated with time since migration. Insofar as migration to Almaty is accompanied by improvements in affordability, desirability or availability of *healthy* foods, these factors may have plausibly contributed to observed improvements in diet quality despite declines in nutrition knowledge.

To the extent that shifting DoFC collectively represents a measure of dietary acculturation, stable metabolic health observed among migrants to Almaty, but not Ulaanbaatar, may be partly explained not only by exposure to healthier food and civic environments but by active acculturation to healthier dietary habits therein. Factor loadings for acculturated diet patterns in Almaty and Ulaanbaatar were negative for seven and one unhealthy food group(s), respectively, and PDQS scores were higher among migrants to Almaty than Ulaanbaatar at all four assessments. Significant, positive, multivariable-adjusted associations between nutrition knowledge and adherence to acculturated patterns were observed in both cities, and while the extent to which acculturation leads to healthier diets is context-specific, positive associations between nutrition knowledge and diet quality were also observed in both cities despite substantial differences in migration dynamics, food environments and trends in nutrition knowledge and diet quality themselves. This may implicate objective nutrition knowledge as a partial proxy for acculturative stress and reinforce nutrition knowledge as a modifiable factor in enabling migrants to adopt healthy dietary habits while navigating urban food environments.

This study was strengthened by concurrent assessment of diverse domains of demographics, nutrition status, lifestyle risk factors, DoFC and nutrition knowledge that provided nuanced perspectives on food choice decisions and their objective and subjective correlates. The use of harmonised assessment methods enabled direct comparisons between cities in different countries, and a panel design involving four repeated measures allowed precise estimation of within-person changes in assessed parameters. By restricting to recent, first-time migrants and using a mixed-effects modelling approach, we could ensure participants were relatively unacculturated at baseline and a broad distribution of times since migration could contribute to the analysis, respectively.

However, because follow-up began after participants had moved to each city, it was impossible to measure more momentous changes occurring during migration events *per se*. Partly for this reason, the sampling approach prioritised the number of repeated measurements over that of unique participants to provide adequate power for the primary aim of capturing within-person changes. This trade-off decreased our power for exploring associations between concurrent trends within cities, precluded inclusion of non-migrant controls and limited the extent to which our results are generalisable to two very large and heterogeneous target populations (which we were not positioned to compare statistically). Limited generalisability may be particularly true in the case of Almaty, where a sample frame was not defined and in which respondent-driven sampling could have also contributed to selection bias. Furthermore, diet was assessed using a food group-based screener that, while rapidly administered and readily analysable for understanding diet quality, prevented analysis of nutrient intakes and reduced resolution with which dietary patterns and trends could be captured. Generally, because most assessments were subjective in nature, they were varyingly influenced by learning effects over repeat assessments, social desirability and other forms of participation bias. Finally, given the large number of statistical tests conducted, some were likely significant by chance. Overall, findings should be interpreted with caution, considering how trends track with one another within cities and qualitatively compare between cities and with the understanding that hypotheses generated from this largely exploratory study are primarily intended to provide a foundation for guiding more focused evaluations going forward.

### Implications

Findings from this study should guide focused efforts to map peri-urban food environments in Ulaanbaatar, drawing on a local history of spatial participatory and sustainability research^(43–45)^, to advocate evidence-based strategies for empowering urban migrants to translate nutrition knowledge and dietary guidance towards healthier diets. Prior studies have identified non- and anti-obesogenic ‘transitional’^(15)^ and ‘healthy’^(46)^ Mongolian diet patterns, respectively, which prevail in urban host populations and provide entry points for designing and advocating food-based programmes. Given the vastness of Ulaanbaatar’s peri-urban slum districts (where over one-third of Mongolia’s population lives), effective policies will primarily be implemented through long-term, muti-sectoral poverty reduction, urban planning and community engagement programmes^(40,47–49)^, and research and advocacy should be framed in the context of development priorities to effectively complement these programmes.

Given the observed association between nutrition knowledge and diet quality, circumstantial decline in nutrition knowledge and otherwise dynamic DoFC among migrants to Almaty, findings from this study should be used to inform a focused evaluation to holistically understand these dynamics and distinguish the contributions of different aspects of food-related perceptions and behaviours on diet quality in Almaty migrants. This effort should be guided by contextual food systems research^(50,51)^ and, in turn, guide health promotion and education interventions for disseminating nutrition information and enabling its uptake by urban migrants, ideally employing staged designs based on lengths of residence in the city^(52)^. Broadly, these programmes should support underdeveloped national policy objectives for improving nutrition awareness, quality of the food supply and dietary surveillance^(17,53,54)^ to incentivize concerted, cost-effective non-communicable disease strategy in Kazakhstan^(55)^.

## Supporting information

Bromage et al. supplementary material 1 Bromage et al. supplementary material

Bromage et al. supplementary material 2 Bromage et al. supplementary material

## Supplementary material

For supplementary material/s referred to in this article, please visit https://doi.org/10.1017/S136898002400243X
