# Supplementary material for: Longitudinal analysis of lifestyle risk factors, nutrition status and drivers of food choice among urban migrants in Ulaanbaatar, Mongolia, and Almaty, Kazakhstan: a formative study
Source: Public Health Nutr. 2024 Dec 3;28(1):e33. doi: 10.1017/S136898002400243X (PMC11825362; doi:10.1017/S136898002400243X)
Supplement: Bromage et al. supplementary material 1 — Bromage et al. supplementary material [file S136898002400243Xsup001.pdf]

Supplementary Methods 1: Summary of questionnaire modules

Eight modules were assessed in the following sequence:

(1) Demographic characteristics, household structure, and household migration history.

(2) Dietary habits, measured in terms of the frequency of consumption of key nutritionally relevant food groups included in the Prime Diet Quality Score (PDQS)[1], a holistic metric of diet quality designed for use in diverse populations that we operationalized as a screening instrument by adapting published guidance (**Methods S2**).

(3) Drivers of food choice (evaluating the subjective importance of diverse considerations in food choice decisions and related perceptions and behaviors, adapting a constructionist food choice process model and framework for understanding internal and external domains of food environments)[2,3].

(4) Nutrition knowledge (evaluating participants' objective understanding of the relative nutritiousness of different food groups and application of nutrition knowledge in daily life, adapting FAO methodology)[4].

(5) International Physical Activity Questionnaire-Short Form (IPAQ-SF)[5] analyzed using an Excel-based system[6].

(6) Pittsburgh Sleep Quality Index (PSQI)[7] (higher scores indicate poorer sleep quality).

(7) Generalized Anxiety Disorder 7-Item (GAD-7) Scale[8] (higher scores indicate a greater degree of generalized anxiety).

(8) Fagerström Test for Nicotine Dependence[9] (higher scores indicate more intense physical addition to nicotine).

The IPAQ, PSQI, GAD-7, and Fagerström Test have been extensively validated in diverse international contexts and all four of these instruments and metrics have been used in prior peer-reviewed studies in Mongolia and Kazakhstan based on a recent Google Scholar search). While the PDQS is a relatively new metric, it has also been widely applied and validated, including as part of research to develop the closely related Global Diet Quality Score (GDQS)[10] which involved analysis of over 65,000 adults in numerous world regions.

Reference periods over which different sets of questions were asked varied from "prior to migration", "currently" or undefined, "past 2 weeks", "past month", to "past 3 months" to increase interpretability of questions and relevance of responses. Questions regarding dietary habits and nutrition knowledge were asked in reference to the past 3 months, such that the "combined" reference period for these questions across the four assessments ranged 12 months, i.e., from 3 months prior to migration to baseline (assessed at the baseline) to 6-9 months post-migration (assessed at 9 months).

An English version of the questionnaire used in this study is provided in **Methods S3**.

References

1. Fung TT, Isanaka S, Hu FB *et al*. International food group-based diet quality and risk of coronary heart disease in men and women. Am J Clin Nutr 2018;107(1):120–9.
2. Sobal J, Bisogni CA. Constructing food choice decisions. Annals of behavioral medicine. 2009 Dec 1;38(suppl_1):s37-46.
3. Turner C, Aggarwal A, Walls H *et al*. Concepts and critical perspectives for food environment research: a global framework with implications for action in low-and middle-income countries. Glob food sec. 2018 Sep 1;18:93-101.
4. Marías YF, Glasauer P. Guidelines for assessing nutrition-related knowledge, attitudes and practices. Food and Agriculture Organization of the United Nations (FAO). Rome FAO; 2014.
5. Craig C, Marshall A, Sjostrom M *et al*. International physical activity questionnaire-short form. J Am Coll Health. 2017;65(7):492-501.

6. Cheng HL. A simple, easy-to-use spreadsheet for automatic scoring of the International Physical Activity Questionnaire (IPAQ) Short Form. 2016. DOI: 10.13140/RG.2.2.21067.80165.
7. Buysse DJ, Reynolds CF 3rd, Monk TH *et al*. The Pittsburgh Sleep Quality Index: a new instrument for psychiatric practice and research. Psychiatry Res. 1989 May;28(2):193-213.
8. Spitzer RL, Kroenke K, Williams JB *et al*. A brief measure for assessing generalized anxiety disorder: the GAD-7. Arch Intern Med. 2006 May 22;166(10):1092-7.
9. Heatherton TF, Kozlowski LT, Frecker RC *et al*. The Fagerström test for nicotine dependence: a revision of the Fagerström Tolerance Questionnaire. Br J Addict. 1991 Sep;86(9):1119-27.
10. Bromage S, Batis C, Bhupathiraju SN *et al*. Development and validation of a novel food-based Global Diet Quality Score (GDQS). J Nutr. 2021 Oct;151(Supplement_2):75S-92S.

Supplementary Methods 2: Operationalizing the PDQS metric for use as a dietary screener

The following methods were implemented in consultation with the developers of the PDQS and GDQS, with the understanding that it is appropriate to carefully adapt the design of these and other metrics of diet quality and dietary diversity, and the ways they are operationalized as dietary screening instruments, to suit the needs of different studies and contexts. Adaptations were also influenced by the fact that design and implementation of the current study occurred during a period in which the initial version of the PDQS[1] underwent iterative refinements which later led to the development of the first PDQS-based screening instrument[2] and the GDQS[3].

List of food groups: To collectively operationalize PDQS food groups as a screening instrument, each group was described using local example foods (selected in consultation with dieticians in Ulaanbaatar and Almaty) adapting published guidance[2]. All of the food groups included in the most recent published version of the PDQS metric[2] were represented in the screener. The following adjustments were made for local interpretability (as informed by the pilot test) and in consultation with the developers of the PDQS and GDQS.

- Fried foods were restricted to those obtained outside the home and deep orange fruits and vegetables were combined into one food group (as has been done in original and subsequent versions of the PDQS, respectively)[1,4].

- Sugar-sweetened beverages were expanded to include (non-homemade) fruit juice, considering evidence from large, prospective cohort studies on associations between consumption of fruit juice, weight gain, and incident type 2 diabetes which informed inclusion of fruit juice in the GDQS[3].

- Low fat milk and dairy products (consumption of which were understood to be relatively low in both cities)[5,6] were excluded and replaced with any milk and dairy (mostly representing high fat milk and dairy, which is also included in GDQS)[3].

- Some adjustments were also made to the names of food groups for local interpretability.

Game meat and shellfish (which are represented in the GDQS)[3] were not considered for inclusion in the screener because consensus on the importance of these foods to global diets and nutrition had not been reached by the GDQS development team until after implementation of the current study had begun. However, these foods were understood to be minor contributors to the diets of urban populations in landlocked Mongolia and Kazakhstan as informed by our prior research in Mongolia[19] and in consultation with the research team at the Kazakh Academy of Nutrition.

Frequency response options: The range of PDQS frequency response options was expanded (as has been done in other frequency-based PDQS screening instruments)[2] from 0-1 per week, 2-3 per week, and $\geq$4 per week to <1 per week, 1 per week, 2-4 per week, 5-7 per week, and >1 per day. This approach was more easily interpreted in the pilot test, and provided greater resolution for understanding consumption patterns and deriving exploratory diet patterns (which were objectives of particular interest in this study).

References

1. Fung TT, Isanaka S, Hu FB *et al*. International food group-based diet quality and risk of coronary heart disease in men and women. Am J Clin Nutr 2018;107(1):120–9.
2. Kronsteiner-Gicevic S, Mou Y, Bromage S *et al*. Development of a Diet Quality Screener for Global Use: Evaluation in a Sample of US Women. J Acad Nutr Diet. 2021 May;121(5):854-871.e6.
3. Bromage S, Batis C, Bhupathiraju SN *et al*. Development and validation of a novel food-based Global Diet Quality Score (GDQS). J Nutr. 2021 Oct;151(Supplement_2):75S-92S.
4. Gicevic S, Gaskins AJ, Fung TT *et al*. Demographic and socio-economic predictors of diet quality among adults in Bosnia and Herzegovina. Public Health Nutr 2019;22(17):3107–17.
5. Bromage S, Daria T, Lander RL *et al*. Diet and Nutrition Status of Mongolian Adults. Nutrients. 2020 May 22;12(5):1514.
6. Euromonitor International. Drinking Milk Products in Kazakhstan. Report code: ASDR-338877. London: Euromonitor; Oct 2020.

Supplementary Methods 3: English version of the questionnaire used in this study (8 pages)

I. Demographics

1. Today's date (DD/MM/YY) ______/______/______

2. Subject code ___________________________

Baseline Questions (skip during follow-up visits)

3. Sex: ◯ **a.** Male ◯ **b.** Female **4.** Date of birth (DD/MM/YY) ______/______/______ **5.** Ethnicity________________

6. Highest education level
◯ **a.** None ◯ **b.** Primary school ◯ **c.** Secondary school ◯ **d.** Professional certificate
◯ **e.** High school ◯ **f.** University ◯ **g.** Graduate degree ◯ **h.** Postgraduate degree

7. Occupation prior to migration ______________ **8. D**welling prior to migration: ◯ **a.** Ger ◯ **b.** House ◯ **c.** Apartment

9. When did you migrate to Ulaanbaatar/Almaty? (DD/MM/YY) ______/______/______

Where did you migrate from? **10.** Aimag/Oblast: ______________ **11.** Soum/Rayon: ______________
12. ◯ **a.** Countryside/Rural village ◯ **b.** Soum/Rayon center ◯ **c.** Aimag/Oblast center

13. What was the purpose of your migration? ____

14. Had you lived in Ulaanbaatar/Almaty previously? ◯ **a.** No ◯ **b.** Yes

15. How long will you reside in Ulaanbaatar/Almaty? ◯ **a.** Less than one year ◯ **b.** One year or more

Who lived in your household prior to migration? (relationship to you and age in years)
16. Relationship_________________________ **17.** Age_____ **18.** Relationship_________________________ **19.** Age_____
20. Relationship_________________________ **21.** Age_____ **22.** Relationship_________________________ **23.** Age_____
24. Relationship_________________________ **25.** Age_____ **26.** Relationship_________________________ **27.** Age_____
28. Relationship_________________________ **29.** Age_____ **30.** Relationship_________________________ **31.** Age_____

Who migrated with you to Ulaanbaatar/Almaty?
32. Relationship_________________________ **33.** Age_____ **34.** Relationship_________________________ **35.** Age_____
36. Relationship_________________________ **37.** Age_____ **38.** Relationship_________________________ **39.** Age_____
40. Relationship_________________________ **41.** Age_____ **42.** Relationship_________________________ **43.** Age_____
44. Relationship_________________________ **45.** Age_____ **46.** Relationship_________________________ **47.** Age_____

Who do you currently live with in Ulaanbaatar/Almaty?
48. Relationship_________________________ **49.** Age_____ **50.** Relationship_________________________ **51.** Age_____
52. Relationship_________________________ **53.** Age_____ **54.** Relationship_________________________ **55.** Age_____
56. Relationship_________________________ **57.** Age_____ **58.** Relationship_________________________ **59.** Age_____
60. Relationship_________________________ **61.** Age_____ **62.** Relationship_________________________ **63.** Age_____

Follow-up Questions (skip during baseline visit)

64. Current Height (cm) ________ **65.** Current Weight (kg) ________

66. What is your current occupation in Ulaanbaatar/Almaty?____

67. Type of dwelling in Ulaanbaatar/Almaty: ◯ **a.** Ger ◯ **b.** House ◯ **c.** Apartment

Who do you currently live with in Ulaanbaatar/Almaty? (relationship to you and age in years)
68. Relationship_________________________ **69.** Age_____ **70.** Relationship_________________________ **71.** Age_____
72. Relationship_________________________ **73.** Age_____ **74.** Relationship_________________________ **75.** Age_____
76. Relationship_________________________ **77.** Age_____ **78.** Relationship_________________________ **79.** Age_____
80. Relationship_________________________ **81.** Age_____ **82.** Relationship_________________________ **83.** Age_____

II. Dietary habits

1. Subject code ________________ **2.** Today's date (DD/MM/YY) ______/______/______

How often, on average, have you used each food group **over the past 3 months**? Mark **one and only one** circle for each food group. For example: If you have usually eaten fish three times a week, please fill in “2-4 per week".

FOOD GROUP	A<br>**<1 per week**	B<br>**1 per week**	C<br>**2-4 per week**	D<br>**5-7 per week**	E<br>**>1 per day**
**3. RED MEAT.** *Beef, sheep, goat, pig, horse, camel. Includes organs. Excludes processed meat.*	◯	◯	◯	◯	◯
**4. PROCESSED MEAT.** *E.g. sausage, luncheon meat, bacon, pâté, chicken nuggets.*	◯	◯	◯	◯	◯
**5. POULTRY.** *E.g. chicken, turkey, game birds. Excludes organs, chicken nuggets, lunch meat, pâté.*	◯	◯	◯	◯	◯
**6. FISH.** *Includes canned, smoked, preserved fish. Excludes shellfish, other seafood.*	◯	◯	◯	◯	◯
**7. MILK & MILK PRODUCTS.** *Includes liquid, soft, hard, powdered milk products. Excludes ice cream.*	◯	◯	◯	◯	◯
**8. EGGS.** *Boiled eggs, fried eggs, poached eggs, scrambled eggs, omelets, raw yolks.*	◯	◯	◯	◯	◯
**9. FRIED FOODS OUTSIDE HOME.** *E.g. fried fast food, street food, restaurant food eaten outside home.*	◯	◯	◯	◯	◯
**10. REFINED GRAINS & PRODUCTS.** *White rice; refined grain porridge, bread, noodles, biscuits.*	◯	◯	◯	◯	◯
**11. POTATOES & OTHER WHITE TUBERS.** *Includes mashed potatoes, potato salad, homemade fries.*	◯	◯	◯	◯	◯
**12. SWEETS & ICE CREAM.** *E.g. candy, candy bar, chocolate, chocolate biscuit, wafer, cake, ice cream.*	◯	◯	◯	◯	◯
**13. SUGARY DRINKS.** *Soda, juice, sweet tea, energy & sports drinks. Excludes homemade juice, dairy.*	◯	◯	◯	◯	◯
**14. CITRUS FRUITS.** *E.g. orange, lemon, mandarin, tangerine. Excludes juice, nectar, fruit sauce, jam.*	◯	◯	◯	◯	◯
**15. DEEP ORANGE FRUITS & VEGETABLES.** *E.g. apricot, mango, pumpkin, carrot, carrot salad.*	◯	◯	◯	◯	◯
**16. OTHER FRUITS.** *Includes dried & preserved fruits, fruit salad. Excludes juice, nectar, fruit sauce, jam.*	◯	◯	◯	◯	◯
**17. DARK GREEN LEAFY VEGETABLES.** *E.g. spinach, lettuce, collards, bok choi, arugula, seaweed.*	◯	◯	◯	◯	◯
**18. CRUCIFEROUS VEGETABLES.** *E.g. broccoli, cabbage, cabbage salad, kimchi, radish.*	◯	◯	◯	◯	◯
**19. OTHER VEGETABLES.** *E.g. pepper, onion, cucumber, tomato, beetroot, eggplant. Excludes legumes.*	◯	◯	◯	◯	◯
**20. LEGUMES.** *Beans, peas, lentils. Includes bean products (e.g. tofu). Excludes nuts and seeds.*	◯	◯	◯	◯	◯
**21. NUTS & SEEDS.** *E.g. sunflower seeds, pine nuts, peanuts, pistachios. Includes peanut butter.*	◯	◯	◯	◯	◯
**22. WHOLE GRAINS & PRODUCTS.** *Brown rice, millet; whole grain cereal, porridge, bread, noodles.*	◯	◯	◯	◯	◯
**23. LIQUID OILS.** *Liquid vegetable oils for cooking & eating raw. E.g. sunflower oil, vegetable oil, olive oil.*	◯	◯	◯	◯	◯

III. Drivers of food choice

1. Subject code ________________ **2.** Today's date (DD/MM/YY) ______/______/______

Compared with **unhealthy** foods, **healthy** foods are generally...

3.
a. ◯ Much less expensive
b. ◯ Less expensive
c. ◯ The same price
d. ◯ More expensive
e. ◯ Much more expensive

4.
a. ◯ Much less available
b. ◯ Less available
c. ◯ Equally available
d. ◯ More available
e. ◯ Much more available

5.
a. ◯ Much less tasty
b. ◯ Less tasty
c. ◯ Equally tasty
d. ◯ Tastier
e. ◯ Much tastier

6.
a. ◯ Much harder to cook
b. ◯ Harder to cook
c. ◯ Same difficulty
d. ◯ Easier to cook
e. ◯ Much easier to cook

For each statement, indicate whether you *"Strongly Disagree", "Disagree",* are *"Neutral", "Agree",* or *"Strongly Agree".*

	**a.** Strongly Disagree	**b.** Disagree	**c.** Neutral	**d.** Agree	**e.** Strongly Agree
**7.** "I care about eating healthy foods."	◯	◯	◯	◯	◯
**8.** "My nutrition knowledge helps me to make healthier food choices."	◯	◯	◯	◯	◯
**9.** "I pay attention to nutrition information on food packaging."	◯	◯	◯	◯	◯
**10.** "I am capable of cooking healthy foods."	◯	◯	◯	◯	◯
**11.** "I eat worse when I am stressed, depressed, or tired."	◯	◯	◯	◯	◯

Describe the influence that each has had on your food choices **over the past 3 months** as:
"None", "Weak", "Moderate", "Strong", or *"Very Strong".*

	**a.** None	**b.** Weak	**c.** Moderate	**d.** Strong	**e.** Very Strong
**12.** The price of food	◯	◯	◯	◯	◯
**13.** The availability of food where you live	◯	◯	◯	◯	◯
**14.** The taste of food	◯	◯	◯	◯	◯
**15.** The time, effort, or skill required to cook food	◯	◯	◯	◯	◯
**16.** The nutritive quality of food	◯	◯	◯	◯	◯
**17.** Public advisories, TV, radio, or web programs	◯	◯	◯	◯	◯
**18.** Information from medical professionals	◯	◯	◯	◯	◯
**19.** Food safety concerns	◯	◯	◯	◯	◯

20. Compared with foods cooked **at home**, foods **from outside** are generally...
◯ Much Less Healthy ◯ Less Healthy ◯ Equally Healthy ◯ Healthier ◯ Much Healthier

21. Overall, **over the past 3 months**, food advertising has influenced me to eat...
◯ Much Less Healthy ◯ Less Healthy ◯ No Influence ◯ Healthier ◯ Much Healthier

For each statement, indicate whether you *"Strongly Disagree", "Disagree",* are *"Neutral", "Agree",* or *"Strongly Agree".*

"My diet influences my..."

	**a.** Strongly Disagree	**b.** Disagree	**c.** Neutral	**d.** Agree	**e.** Strongly Agree
**22.** Health	◯	◯	◯	◯	◯
**23.** Physical fitness	◯	◯	◯	◯	◯
**24.** Physical appearance	◯	◯	◯	◯	◯
**25.** Bodyweight	◯	◯	◯	◯	◯
**26.** Household members' diets	◯	◯	◯	◯	◯

IV. Nutrition knowledge

1. Subject code _________________ **2.** Today's date (DD/MM/YY) ______/______/______

Over the past **3 months**...

3. ...how would you describe your diet overall?
a. ◯ Very Healthy
b. ◯ Healthy
c. ◯ Average
d. ◯ Unhealthy
e. ◯ Very Unhealthy

4. ...how would you describe your nutrition knowledge?
a. ◯ Well Informed
b. ◯ Informed
c. ◯ Average
d. ◯ Uninformed
e. ◯ Very Uninformed

5. ...how frequently has your household cooked its meals?
a. ◯ All Meal
b. ◯ Most Meals
c. ◯ Some Meals
d. ◯ Few Meals
e. ◯ No Meals

6. ...how would you characterize your own cooking skills?
a. ◯ Very Skilled
b. ◯ Skilled
c. ◯ Average
d. ◯ Poor
e. ◯ Very Poor

7. ...how often has your household eaten together
a. ◯ All Meal
b. ◯ Most Meals
c. ◯ Some Meals
d. ◯ Few Meals
e. ◯ No Meals
f. ◯ Not Applicable (single-person household)

8. ...how would you describe your bodyweight?
a. ◯ Overweight
b. ◯ Normal
c. ◯ Underweight

9. ...how would you describe your physical activity level?
a. ◯ High
b. ◯ Moderate
c. ◯ Low

Check which among the following pairs of foods is generally more nutritious for **healthy adults** to eat **habitually** (if you are unsure or don't know, check that option):

10. a. ◯ Red Meat (mutton, beef, pork)
b. ◯ Lean Meat (chicken, fish)
c. ◯ Unsure

11. a. ◯ Whole Fat Milk and Dairy Products
b. ◯ Reduced Fat Milk and Dairy Products
c. ◯ Unsure

12 a. ◯ Cooking Oils (sunflower seed oil, other oils)
b. ◯ Cooking Fats (solid animal fats, butter)
c. ◯ Unsure

13. a. ◯ Whole Grains (brown flour/bread/noodles/rice)
b. ◯ Refined Grains (white flour/bread/noodles/rice)
c. ◯ Unsure

Check whether it is generally more nutritious for **healthy adults** to eat more or less of each of the following foods **habitually** (if you are unsure or don't know, check that option):

	**a.** More	**b.** Less	**c.** Unsure
**14.** Salt and Salty Foods:	◯	◯	◯
**15.** Sugar and Sugary Foods/Drinks:	◯	◯	◯
**16.** Fruits and Vegetables:	◯	◯	◯
**17.** Nuts and Seeds:	◯	◯	◯
**18.** Processed and Fast Foods:	◯	◯	◯
**19.** Alcoholic Drinks:	◯	◯	◯

V. Physical activity

1. Subject code _____________ **2.** Today's date (DD/MM/YY) ______/______/______

We are interested in finding out about the kinds of physical activities that people do as part of their everyday lives. The questions will ask you about the time you spent being physically active in the **last 1 month**. Please answer each question even if you do not consider yourself to be an active person. Please think about the activities you do at work, as part of your house and yard work, to get from place to place, and in your spare time for recreation, exercise or sport.

Think about all the **vigorous** activities that you did in the **last 1 month**. **Vigorous** physical activities refer to activities that take hard physical effort and make you breathe much harder than normal. Think only about those physical activities that you did for at least 10 minutes at a time.

3. During the last **1 month**, on how many days did you do **vigorous** physical activities like heavy lifting, digging, aerobics, or fast bicycling?

a. _____ days per week

b. ◯ No **vigorous** physical activities (*skip to question 5)*

4. How much time did you usually spend doing **vigorous** physical activities on one of those days?

a. _____ hours and _____ minutes per day

b. Don't know/Not sure

Think about all the **moderate** activities that you did in the **last 1 month**. **Moderate** activities refer to activities that take moderate physical effort and make you breathe somewhat harder than normal. Think only about those physical activities that you did for at least 10 minutes at a time.

5. During the **last 1 month**, on how many days did you do **moderate** physical activities like carrying light loads, bicycling at a regular pace, or doubles tennis? Do not include walking.

a. _____ days per week

b. ◯ No **moderate** physical activities (*skip to question 7)*

6. How much time did you usually spend doing **moderate** physical activities on one of those days?

a._____ hours and _____ minutes per day

b. Don't know/Not sure

Think about the time you spent **walking** in the **last 1 month**. This includes at work and at home, walking to travel from place to place, and any other walking that you have done solely for recreation, sport, exercise, or leisure.

7. During the **last 7 days**, on how many days did you **walk** for at least 10 minutes at a time?

a. _____ days per week

b. No **walking** (*skip to question 9)*

8. How much time did you usually spend **walking** on one of those days?

a._____ hours and _____ minutes per day

b. Don't know/Not sure

The last question is about the time you spent **sitting** on weekdays during the **last 1 month**. Include time spent at work, at home, while doing course work and during leisure time. This may include time spent sitting at a desk, visiting friends, reading, or sitting or lying down to watch television.

9. During the **last 1 month**, how much time did you spend **sitting** on a **week day**?

a. _____ hours and _____ minutes per day

b. Don't know/Not sure

VI. Sleep quality

1. Subject code ________________ **2.** Today's date (DD/MM/YY) ______/______/______

The following questions relate to your usual sleep habits during the **past month** only. Your answers should indicate the most accurate reply for the majority of days and nights in the **past month**. Please answer all questions.

During the **past month**...

3. When have you usually gone to bed? ________________________________
4. How long (in minutes) has it taken you to fall asleep each night? ________________________________
5. What time have you usually gotten up in the morning? ________________________________
6. A. How many hours of actual sleep did you get at night? ________________________________
7. How many hours were you in bed?

During the **past month**, how often have you had trouble sleeping because you

	**a.** Not during the past month	**b.** Less than once a week	**c.** Once or twice a week	**d.** Three or more times a week
**8.** Cannot get to sleep within 30 minutes	◯	◯	◯	◯
**9.** Wake up in the middle of the night or early morning	◯	◯	◯	◯
**10.** Have to get up to use the bathroom	◯	◯	◯	◯
**11.** Cannot breathe comfortably	◯	◯	◯	◯
**12.** Cough or snore loudly	◯	◯	◯	◯
**13.** Feel too cold	◯	◯	◯	◯
**14.** Feel too hot	◯	◯	◯	◯
**15.** Have bad dreams	◯	◯	◯	◯
**16.** Have pain	◯	◯	◯	◯
**17.** Other reason (s), please describe, including how often you have had trouble sleeping because of this reason (s):	◯	◯	◯	◯

18. During the **past month**, how often have you taken medicine (prescribed or “over the counter”) to help you sleep?
a. ◯ Not during the past month
b. ◯ Less than once a week
c. ◯ Once or twice a week
d. ◯ Three or more times a week

19. During the **past month**, how often have you had trouble staying awake while driving, eating meals, or engaging in social activity?
a. ◯ Not during the past month
b. ◯ Less than once a week
c. ◯ Once or twice a week
d. ◯ Three or more times a week

20. During the **past month**, how much of a problem has it been for you to keep up enthusiasm to get things done?
a. ◯ Not during the past month
b. ◯ Less than once a week
c. ◯ Once or twice a week
d. ◯ Three or more times a week

21. During the **past month**, how would you rate your sleep quality overall?
a. ◯ Very good
b. ◯ Fairly good
c. ◯ Fairly bad
d. ◯ Very bad

VII. Generalized anxiety

1. Subject code __________________ **2.** Today's date (DD/MM/YY) ______/______/______

Over the past **2 weeks**, how often have you been bothered by the following problems?

	**a.** Not at all sure	**b.** Several days	**c.** Over half the days	**d.** Nearly every day
**1.** Feeling nervous, anxious, or on edge	◯	◯	◯	◯
**2.** Not being able to stop or control worrying	◯	◯	◯	◯
**3.** Worrying too much about different things	◯	◯	◯	◯
**4.** Trouble relaxing	◯	◯	◯	◯
**5.** Being so restless that it's hard to sit still	◯	◯	◯	◯
**6.** Becoming easily annoyed or irritable	◯	◯	◯	◯
**7.** Feeling afraid as if something awful might happen	◯	◯	◯	◯

VIII. Nicotine dependence

1. Subject code __________________ **2.** Today's date (DD/MM/YY) ______/______/______

3. How soon after waking do you smoke your first cigarette?
a. ◯ Within 5 minutes
b. ◯ 5-30 minutes
c. ◯ 31-60 minutes

4. Do you find it difficult to refrain from smoking in places where it is forbidden? E.g. church, library, etc.
a. ◯ Yes
b. ◯ No

5. Which cigarette would you hate to give up?
a. ◯ The first in the morning
b. ◯ Any other

6. How many cigarettes a day do you smoke?
a. ◯ 10 or less
b. ◯ 11-20
c. ◯ 21-30
d. ◯ 31 or more

7. Do you smoke more frequently in the morning?
a. ◯ Yes
b. ◯ No

8. Do you smoke even if you are sick in bed most of the day?
a. ◯ Yes
b. ◯ No

Supplementary Methods 4: Approach for scoring the PDQS and PDQS sub-metrics

The dietary screener was used to score the PDQS metric by adapting scoring principles described in published guidance[1] to the range of consumption frequency options adapted for use in this study: healthy food groups were assigned 0, 1, 2, 3, or 4 points for responses of <1 per week, 1 per week, 2-4 per week, 5-7 per week, and >1 per day, respectively; scoring was reversed for unhealthy food groups; and the total score ranged from 0 to 80. “PDQS-healthy” and “PDQS-unhealthy” sub-metrics were also scored using only the healthy and unhealthy PDQS food groups, respectively, following principles for scoring the positive and negative sub-metrics of the related Global Diet Quality Score (GDQS)[2] (as with the GDQS sub-metrics, higher PDQS-healthy scores reflect higher consumption of healthy foods while higher PDQS-unhealthy scores reflect lower consumption of unhealthy foods).

The approach for classifying healthy and unhealthy PDQS food groups followed that which has been applied in published guidance[1,3-5], with the exception of eggs. Eggs, which the PDQS has varyingly classified as a neutral or healthy food group[1-5], were classified as a healthy group considering evolving evidence from large, prospective cohort studies on the relationship between egg consumption and health that informed inclusion of eggs in the GDQS[2].

Further, despite updated evidence that informed reclassification of red meat and high-fat milk and dairy products from the PDQS (which considers these to be unhealthy and neutral dietary components, respectively, for adults)[1] to the GDQS (which considers both components to be unhealthy only when consumed in excessive amounts)[2], we retained the classifications used in the PDQS, considering that:

- Red meat is consumed in extremely high amounts in the Mongolian and Kazakhstan general populations[6,7] and it was assumed that habitual consumption by most participants could be reasonably classified as excessive (defined according to the GDQS as >46 g/day).

- High-fat milk and dairy products are also consumed in large amounts in the general population of both countries[6-8]. However, despite having expanded the range of PDQS frequency response options, the maximum consumption frequency captured by the screening instrument developed for use in this study was >1 per day, while the GDQS defines excessive consumption of high fat milk and dairy as >734 g/day (roughly equivalent to >3 servings or milk equivalents). This reasonably precluded adapting the GDQS scoring approach for high fat milk and dairy products in this study.

The final approach for scoring the PDQS and PDQS sub-metrics in this study is summarized below:

Food groups		Point values associated with each frequency response				
		**<1/wk**	**1/wk**	**2-4/wk**	**5-7/wk**	**>1/d**
**Healthy** food group**s** (included in the **PDQS** metric and **PDQS-healthy** sub-metric)	Citrus fruits	0	1	2	3	4
	Other fruits	0	1	2	3	4
	Dark green leafy vegetables	0	1	2	3	4
	Cruciferous vegetables	0	1	2	3	4
	Other vegetables	0	1	2	3	4
	Deep orange fruits and vegetables	0	1	2	3	4
	Legumes	0	1	2	3	4
	Nuts and seeds	0	1	2	3	4
	Whole grains and products	0	1	2	3	4
	Liquid oils	0	1	2	3	4
	Fish	0	1	2	3	4
	Poultry	0	1	2	3	4
	Eggs	0	1	2	3	4
**Unhealthy** food groups (including in the **PDQS** metric and **PDQS-unhealthy** sub-metric)	Red meat	4	3	2	1	0
	Processed meat	4	3	2	1	0
	Refined grains and products	4	3	2	1	0
	Sweets and ice cream	4	3	2	1	0
	Sugary drinks	4	3	2	1	0
	Potatoes and other white tubers	4	3	2	1	0
	Fried foods from outside home	4	3	2	1	0
Not scored	Milk and milk products	0	0	0	0	0

References

1. Fung TT, Isanaka S, Hu FB *et al*. International food group-based diet quality and risk of coronary heart disease in men and women. Am J Clin Nutr 2018;107(1):120–9.
2. Bromage S, Batis C, Bhupathiraju SN *et al*. Development and validation of a novel food-based Global Diet Quality Score (GDQS). J Nutr. 2021 Oct;151(Supplement_2):75S-92S.
3. Kronsteiner-Gicevic S, Mou Y, Bromage S *et al*. Development of a Diet Quality Screener for Global Use: Evaluation in a Sample of US Women. J Acad Nutr Diet. 2021 May;121(5):854-871.e6.
4. Gicevic S, Gaskins AJ, Fung TT *et al*. Demographic and socio-economic predictors of diet quality among adults in Bosnia and Herzegovina. Public Health Nutr 2019;22(17):3107–17.
5. Gicevic S, Tahirovic E, Bromage S *et al*. Diet quality and all-cause mortality among US adults, estimated from NHANES, 2003–2008. Public Health Nutr 2021:24(10):2777–872.
6. Bromage S, Daria T, Lander RL *et al*. Diet and Nutrition Status of Mongolian Adults. Nutrients. 2020 May 22;12(5):1514.
7. Jia M, Zhen L, Xiao Y. Changing Food Consumption and Nutrition Intake in Kazakhstan. Nutrients. 2022 Jan 13;14(2):326.
8. Euromonitor International. Drinking Milk Products in Kazakhstan. Report code: ASDR-338877. London: Euromonitor; Oct 2020.

Supplementary Methods 5: R packages and functions used in this study

Derivation of exploratory diet patterns: *prcomp* function in the *stats* package[1].

Extraction and visualization of results of pattern analysis: *fviz_eig*, *get_pca_var*, and *get_pca_ind* functions in the *factoextra* package[2].

Linear mixed-effects regression models for continuous outcomes and generalized linear mixed models for one binary outcome: *lmer* and *glmer* functions in the *lme4* package[3].

Cumulative link mixed models for ordered categorical outcomes: *clmm* function in the *ordinal* package[4].

References

1. R Core Team. R: A language and environment for statistical computing. Vienna: R Foundation for Statistical Computing; 2022.
2. Kassambara A, Mundt F. factoextra: Extract and Visualize the Results of Multivariate Data Analyses. R package version 1.0.7. 2020.
3. Bates D, Maechler M, Bolker B *et al*. Fitting Linear Mixed-Effects Models Using lme4. J Stat Softw. 2015;67(1):1-48.
4. Christensen RHB. ordinal - Regression Models for Ordinal Data. R package version 2022. 2022.
