# Supplementary material for: Longitudinal analysis of lifestyle risk factors, nutrition status and drivers of food choice among urban migrants in Ulaanbaatar, Mongolia, and Almaty, Kazakhstan: a formative study
Source: Public Health Nutr. 2024 Dec 3;28(1):e33. doi: 10.1017/S136898002400243X (PMC11825362; doi:10.1017/S136898002400243X)
Supplement: Bromage et al. supplementary material 2 — Bromage et al. supplementary material [file S136898002400243Xsup002.pdf]

Supplementary Results: Nonsignificant (*p*>0.1) trends in drivers of food choice and related perceptions and behaviors

		Almaty				Ulaanbaatar			
**Question**	**Response**	**Baseline, n (%)**	**9 months, n (%)**	**OR (95%CI)[1]**	***p*[1]**	**Baseline, n (%)**	**9 months, n (%)**	**OR (95%CI)[1]**	***p*[1]**
Influence of food safety concerns on your food choice	None	3 (3)	3 (3)	1.06 (0.97, 1.15)	0.184	15 (15)	16 (18)	0.97 (0.92, 1.01)	0.141
	Weak	20 (20)	19 (19)			21 (21)	17 (19)		
	Moderate	74 (74)	62 (62)			18 (18)	28 (31)		
	Strong	3 (3)	15 (15)			24 (24)	16 (18)		
	Very strong	0 (0)	1 (1)			22 (22)	13 (14)		
Influence of information from medical professionals on your food choice	None	3 (3)	2 (2)	0.98 (0.91, 1.06)	0.686	16 (16)	25 (28)	0.98 (0.93, 1.02)	0.271
	Weak	18 (18)	28 (28)			29 (29)	19 (21)		
	Moderate	69 (69)	51 (51)			27 (27)	22 (24)		
	Strong	10 (10)	18 (18)			19 (19)	16 (18)		
	Very strong	0 (0)	1 (1)			9 (9)	8 (9)		
Influence of public advisories, TV, radio, or web programs on your food choice	None	11 (11)	8 (8)	1.01 (0.92, 1.11)	0.884	29 (29)	30 (33)	1.00 (0.95, 1.05)	0.929
	Weak	31 (31)	26 (26)			20 (20)	16 (18)		
	Moderate	57 (57)	54 (54)			20 (20)	20 (22)		
	Strong	1 (1)	9 (9)			14 (14)	13 (14)		
	Very strong	0 (0)	3 (3)			17 (17)	11 (12)		
Food advertising has influenced me to eat…	Much less healthy	0 (0)	0 (0)	1.00 (0.99, 1.00)	0.227	11 (11)	11 (12)	1.00 (0.95, 1.05)	0.963
	Less healthy	18 (18)	18 (18)			26 (26)	25 (28)		
	Equally healthy	65 (65)	57 (57)			53 (53)	48 (53)		
	Healthier	9 (9)	21 (21)			8 (8)	4 (4)		
	Much healthier	8 (8)	4 (4)			2 (2)	2 (2)		
Compared with unhealthy foods, healthy foods are generally…	Much less expensive	0 (0)	0 (0)	1.04 (0.97, 1.13)	0.279	6 (6)	6 (7)	1.03 (0.98, 1.08)	0.259
	Less expensive	6 (6)	7 (7)			6 (6)	13 (14)		
	The same price	34 (34)	26 (26)			24 (24)	14 (16)		
	More expensive	43 (43)	50 (50)			51 (51)	43 (48)		
	Much more expensive	17 (17)	17 (17)			13 (13)	14 (16)		
	Much less tasty	1 (1)	3 (3)	1.00 (0.93, 1.08)	0.952	7 (7)	8 (9)	0.96 (0.92, 1.01)	0.111
	Less tasty	8 (8)	8 (8)			14 (14)	17 (19)		

Compared with unhealthy foods, healthy foods are generally…	Equally tasty	43 (43)	41 (41)			24 (24)	26 (29)		
	Tastier	37 (37)	39 (39)			46 (46)	38 (42)		
	Much tastier	11 (11)	9 (9)			8 (8)	1 (1)		
Compared with foods cooked at home, foods from outside are generally…	Much less healthy	3 (3)	2 (2)	1.04 (0.97, 1.11)	0.314	69 (69)	64 (71)	0.97 (0.92, 1.03)	0.312
	Less healthy	43 (43)	36 (36)			18 (18)	15 (17)		
	Equally healthy	45 (45)	43 (43)			2 (2)	3 (3)		
	Healthier	1 (1)	15 (15)			9 (9)	3 (3)		
	Much healthier	8 (8)	4 (4)			2 (2)	5 (6)		
"My diet influences my health."	Strongly disagree	8 (8)	2 (2)	1.02 (0.96, 1.09)	0.473	3 (3)	5 (6)	1.03 (0.98, 1.09)	0.250
	Disagree	8 (8)	10 (10)			3 (3)	5 (6)		
	Neutral	6 (6)	12 (12)			24 (24)	16 (18)		
	Agree	61 (61)	50 (50)			51 (51)	40 (44)		
	Strongly agree	17 (17)	26 (26)			19 (19)	24 (27)		
"My diet influences my physical fitness."	Strongly disagree	1 (1)	0 (0)	1.01 (0.94, 1.09)	0.720	8 (8)	6 (7)	1.00 (0.95, 1.05)	0.949
	Disagree	11 (11)	11 (11)			8 (8)	7 (8)		
	Neutral	13 (13)	19 (19)			18 (18)	12 (13)		
	Agree	66 (66)	51 (51)			48 (48)	46 (51)		
	Strongly agree	9 (9)	19 (19)			18 (18)	19 (21)		
"My diet influences my physical appearance."	Strongly disagree	2 (2)	1 (1)	1.05 (0.98, 1.13)	0.157	9 (9)	8 (9)	0.99 (0.94, 1.03)	0.572
	Disagree	11 (11)	9 (9)			8 (8)	9 (10)		
	Neutral	11 (11)	17 (17)			26 (26)	19 (21)		
	Agree	68 (68)	54 (54)			38 (38)	39 (43)		
	Strongly agree	8 (8)	19 (19)			18 (18)	15 (17)		
"My diet influences my household members' diets."	Strongly disagree	2 (2)	1 (1)	1.00 (0.94, 1.06)	0.991	10 (10)	6 (7)	1.02 (0.97, 1.07)	0.447
	Disagree	21 (21)	18 (18)			9 (9)	3 (3)		
	Neutral	31 (31)	32 (32)			13 (13)	16 (18)		
	Agree	38 (38)	37 (37)			45 (45)	46 (51)		
	Strongly agree	8 (8)	12 (12)			23 (23)	19 (21)		
"I care about eating healthy foods."	Strongly disagree	10 (10)	3 (3)	1.04 (0.97, 1.10)	0.251	65 (66)	1 (1)	0.99 (0.94, 1.05)	0.821
	Disagree	9 (9)	13 (13)			6 (6)	2 (2)		
	Neutral	46 (46)	43 (43)			18 (18)	18 (20)		

	Agree	32 (32)	32 (32)			48 (48)	49 (54)		
	Strongly agree	3 (3)	9 (9)			26 (26)	20 (22)		
"My nutrition knowledge helps me to make healthier food choices."	Strongly disagree	12 (12)	4 (4)	1.03 (0.97, 1.09)	0.341	60 (67)	4 (4)	0.99 (0.94, 1.04)	0.687
	Disagree	8 (8)	10 (10)			19 (21)	10 (11)		
	Neutral	44 (44)	40 (40)			8 (9)	11 (12)		
	Agree	35 (35)	32 (32)			55 (55)	50 (56)		
	Strongly agree	1 (1)	14 (14)			17 (17)	15 (17)		
"I am capable of cooking healthy foods."	Strongly disagree	9 (9)	3 (3)	1.01 (0.95, 1.08)	0.702	4 (4)	3 (3)	1.04 (0.99, 1.09)	0.127
	Disagree	5 (5)	7 (7)			15 (15)	9 (10)		
	Neutral	22 (22)	22 (22)			19 (19)	18 (20)		
	Agree	53 (53)	55 (55)			48 (48)	45 (50)		
	Strongly agree	11 (11)	13 (13)			14 (14)	15 (17)		
How would you describe your nutrition knowledge?	Well informed	17 (17)	25 (25)	0.97 (0.89, 1.05)	0.394	9 (9)	7 (8)	1.01 (0.94, 1.07)	0.880
	Informed	55 (55)	38 (38)			22 (22)	17 (19)		
	Average	26 (26)	32 (32)			56 (57)	59 (66)		
	Uninformed	2 (2)	5 (5)			12 (12)	7 (8)		
	Very uninformed	0 (0)	0 (0)			0 (0)	0 (0)		

Footnote: [1]OR (95%CI) and *p* statistics indicate the age- and sex-adjusted odds ratio and *p* value for the association between a one-month increase in time since migration and each outcome, estimated using cumulative link mixed models (OR that associated with a one-row descent in ordered category presented in the table, e.g., the OR for “Influence of the price of food on your food choices” is that associated with being in either the “Weak” vs. “None” category, “Moderate” vs. “Weak” category, “Strong” vs. “Moderate” category, or “Very strong” vs. “Strong” category).
